# High-throughput microscopy reveals the impact of multifactorial environmental perturbations on colorectal cancer cell growth

**DOI:** 10.1093/gigascience/giab026

**Published:** 2021-04-19

**Authors:** Chun-Te Chiang, Roy Lau, Ahmadreza Ghaffarizadeh, Matthew Brovold, Dipen Vyas, Edwin F Juárez, Anthony Atala, David B Agus, Shay Soker, Paul Macklin, Daniel Ruderman, Shannon M Mumenthaler

**Affiliations:** Lawrence J. Ellison Institute for Transformative Medicine, University of Southern California, Los Angeles, CA 90064, USA; Lawrence J. Ellison Institute for Transformative Medicine, University of Southern California, Los Angeles, CA 90064, USA; Lawrence J. Ellison Institute for Transformative Medicine, University of Southern California, Los Angeles, CA 90064, USA; Wake Forest Institute for Regenerative Medicine, Winston-Salem, NC 27157, USA; Wake Forest Institute for Regenerative Medicine, Winston-Salem, NC 27157, USA; Lawrence J. Ellison Institute for Transformative Medicine, University of Southern California, Los Angeles, CA 90064, USA; Wake Forest Institute for Regenerative Medicine, Winston-Salem, NC 27157, USA; Lawrence J. Ellison Institute for Transformative Medicine, University of Southern California, Los Angeles, CA 90064, USA; Wake Forest Institute for Regenerative Medicine, Winston-Salem, NC 27157, USA; Lawrence J. Ellison Institute for Transformative Medicine, University of Southern California, Los Angeles, CA 90064, USA; Intelligent Systems Engineering, Indiana University, Bloomington, IN 47408, USA; Lawrence J. Ellison Institute for Transformative Medicine, University of Southern California, Los Angeles, CA 90064, USA; Lawrence J. Ellison Institute for Transformative Medicine, University of Southern California, Los Angeles, CA 90064, USA

**Keywords:** colorectal cancer, high-content imaging, liver metastasis, tumor microenvironment

## Abstract

**Background:**

Colorectal cancer (CRC) mortality is principally due to metastatic disease, with the most frequent organ of metastasis being the liver. Biochemical and mechanical factors residing in the tumor microenvironment are considered to play a pivotal role in metastatic growth and response to therapy. However, it is difficult to study the tumor microenvironment systematically owing to a lack of fully controlled model systems that can be investigated in rigorous detail.

**Results:**

We present a quantitative imaging dataset of CRC cell growth dynamics influenced by *in vivo*–mimicking conditions. They consist of tumor cells grown in various biochemical and biomechanical microenvironmental contexts. These contexts include varying oxygen and drug concentrations, and growth on conventional stiff plastic, softer matrices, and bioengineered acellular liver extracellular matrix. Growth rate analyses under these conditions were performed via the cell phenotype digitizer (CellPD).

**Conclusions:**

Our data indicate that the growth of highly aggressive HCT116 cells is affected by oxygen, substrate stiffness, and liver extracellular matrix. In addition, hypoxia has a protective effect against oxaliplatin-induced cytotoxicity on plastic and liver extracellular matrix. This expansive dataset of CRC cell growth measurements under *in situ* relevant environmental perturbations provides insights into critical tumor microenvironment features contributing to metastatic seeding and tumor growth. Such insights are essential to dynamical modeling and understanding the multicellular tumor-stroma dynamics that contribute to metastatic colonization. It also establishes a benchmark dataset for training and testing data-driven dynamical models of cancer cell lines and therapeutic response in a variety of microenvironmental conditions.

## Background

Colorectal cancer (CRC) is the third most deadly cancer in both men and women in the United States [1]. Current treatment strategies include FOLFOX (fluorouracil, leucovorin, and oxaliplatin), FOLFIRI (fluorouracil, leucovorin, and irinotecan), or XELOX (oxaliplatin and capecitabine) with or without molecular targeted drugs [2]. The 5-year survival rate for CRC is 90% if the cancer is diagnosed locally. However, once the disease has spread to distant sites, this rate decreases dramatically to ∼10% [3]. Emerging data, spanning from clinical to laboratory research, highlight that metastatic disease cannot be explained solely by the genetics of the cancer cells; instead, bidirectional interactions with the surrounding microenvironment play a pivotal role in tumor progression [4–7]. Devising innovative ways to treat CRC metastasis must address not only the genetic heterogeneity of the tumor but also its dynamic microenvironment.

Liver is the most common organ of distant metastasis. More than 50% of CRC patients with advanced disease develop liver metastases (LM) [8]. Many such metastases are discovered months or years after initial seeding. Consequently, most patients require treatment for established liver tumor foci. It has been postulated that the high rate of LM is a result of anatomical considerations, with the portal vein draining directly from the colon and upper rectum to the liver [9]. However, increasing evidence has demonstrated that the liver microenvironment is vital in influencing CRC metastasis [10–12]. Low oxygen levels (hypoxia) are a key component in the liver tumor microenvironment (TME) [13]. The oxygen concentration in the portal vein is ∼1% [14]. Poor vascularization in the tumor mass can further promote an oxygen-limited environment and initiate more aggressive phenotypes [15]. Hypoxia can lead to genomic instability by influencing DNA repair pathways [16]. Specifically, hypoxia has been shown to down-regulate the expression of DNA mismatch repair proteins at both transcriptional and translational levels [16]. Alterations in the mismatch repair machinery are known to result in microsatellite instability, which is found in ∼15% of CRC [17].

Altered extracellular matrix (ECM) has also been considered a key feature of the TME [18]. ECM physically supports tissues and provides a substrate for cell adhesion and migration [19]. Cellular interactions with the surrounding ECM can regulate a vast range of biological outcomes including disease progression and drug resistance [20]. Different tissues are known to have distinct ECM molecular compositions and architectures. The complexity of the ECM is not merely biochemical: associated mechanical properties, such as stiffness, can also greatly affect cell proliferation and motility [21]. It is well known that tumor tissue is much stiffer than surrounding normal tissues [22]. Increased tissue stiffness can influence tumor growth, metabolism, invasion, and metastasis and has been demonstrated to play a significant role in disease progression of several solid tumors and to correlate with patient outcomes [23, 24]. A recent study showed that the stiffness of LM is significantly higher than that of primary CRC tumors and that the metastatic stiffness is closely correlated with tissue vascularity [25]. Moreover, adhesive tumor cell interactions with liver cells—particularly endothelial cells and hepatocytes in the sinusoids—have recently been shown to affect metastatic progression [26], as well as chemical communication with stellate, Kupffer, and inflammatory cells [27], illustrating the importance of ECM stiffness as a parameter to consider in studying CRC progression.

The TME is a highly complex system with many factors working in concert. However, traditional biological assays often only examine a single environmental factor in a qualitative way, and thereby lack the ability to recapitulate essential features of metastatic growth. Multicellular computational modeling can provide novel insights to link cancer progression to heterogeneous TME conditions and the dynamical interactions between tumor cells and the resident cell ecosystem [28, 29]. However, predicting the impact of liver microenvironmental manipulations on CRC behavior requires high-quality benchmark datasets to fit model parameters and drive the development of computational models [30], particularly systems that can independently and separately study the role of metastatic tumor cell interactions with hypoxia, the ECM, and resident liver cells. High-content screening (HCS), the application of automated microscopy and image analysis, has been widely used in cell biology and drug development to screen drug compounds for safety and toxicity on human cells *in vitro* [31]. This platform is also well suited for exploring the impact of multiple TME parameters, either individually or simultaneously, on cell behavior [32, 33]. Here we extend the use of our previously established imaging workflow to study the biophysical and biochemical impact of the organ context, specifically the combination of oxygen, stiffness, and liver ECM, on CRC cell growth and response to therapy. After isolating the impact of hypoxia and ECM biomechanics on CRC cell seeding, we can control for these factors and continue high-throughput investigations that isolate and characterize adhesive and other multicellular interactions during metastatic colonization.

## Results

### CRC growth rates under different oxygen tensions

**Figure 1:**
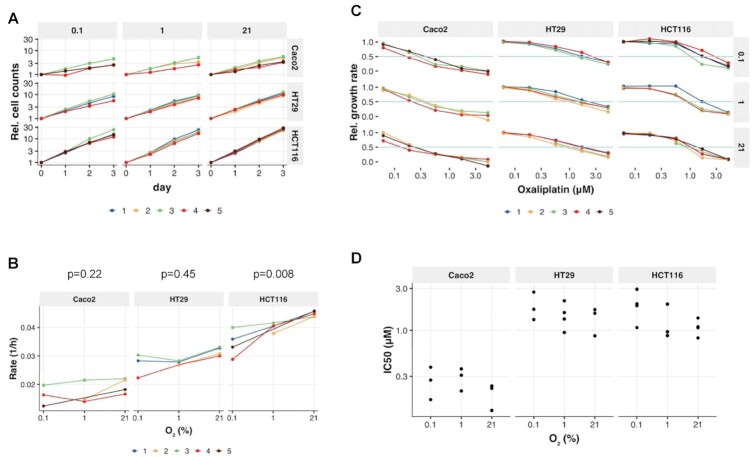
The impact of oxygen on CRC growth and treatment response to oxaliplatin. **A**, Caco2, HT29, and HCT116 cells were cultured in 0.1%, 1%, or 21% oxygen concentration. Cell counts were measured at several time points using Operetta high-content screening platform. **B**, Growth rate of Caco2, HT29, and HCT116 cells in 0.1%, 1%, or 21% oxygen concentration was determined by CellPD. A 2-sided sign test was used to detect instances where all data trended in a single direction. **C**, Relative growth rate of Caco2, HT29, and HCT116 cells treated with 0, 0.062, 0.185, 0.555, 1.667, 5 μM oxaliplatin. **D**, Oxaliplatin IC_50_ changes in 0.1%, 1%, or 21% oxygen environment.

Tumor hypoxia, or oxygen deprivation, has been shown to decrease proliferation and limit cells’ responsiveness to therapeutic agents [34]. To investigate the impact of hypoxia on CRC growth, we examined 3 human CRC cell lines with different aggressiveness (Caco2, HT29, HCT116) grown under various oxygen concentrations (normoxic [21%] and hypoxic [1% and 0.1%]). HCT116 was derived from a poorly differentiated human colon adenocarcinoma known to develop hepatic metastases efficiently in immunodeficient mice. HT29 also has metastatic capabilities but less efficient compared with HCT116. In contrast, Caco2 shows no ability to metastasize [35]. We used the Operetta high-content imaging system and Harmony software (version 3.5.2) to measure cell counts of CRC cell lines grown in the respective oxygen conditions (Fig. 1A and Supplementary Fig. S1). To extract growth rates from sequential cell count data, we used CellPD (cell phenotype digitizer) (version 1.0.1), a previously developed open source Python code that leverages the Levenberg-Marquardt algorithm to perform nonlinear least-squares minimization between simulated and experimental cell counts [36]. We observed a trend toward reduced growth rates under the hypoxic conditions in the most aggressive cell line HCT116 (*P* = 0.008), which is not present in the less aggressive cell lines, HT29 and Caco2 (Fig. 1B).

### The impact of hypoxia on drug response

Oxaliplatin is a standard CRC chemotherapy agent used in the adjuvant and advanced setting [37]. To examine the effect of oxygen on cancer treatment response, we measured CRC growth effects of oxaliplatin under different oxygen concentrations (various concentrations of oxygen: 21%, 1%, 0.1% and oxaliplatin treatment: 0, 0.062, 0.185, 0.555, 1.667, 5 μM). We found the least aggressive cell line, Caco2, to be most sensitive to oxaliplatin treatment compared to HT29 and HCT116 across all oxygen concentrations (Fig. 1C). Interestingly, the IC_50_ of oxaliplatin was not significantly altered under hypoxia in both Caco2 and HT29 cells. However, there was a difference in the IC_50_ of oxaliplatin between 0.1% and 21% oxygen, with a mean estimate of 1.7-fold increase (95% credible interval [95% CI], 1.02–2.97) in HCT116 cells (Fig. 1D).

### Matrix stiffness environment influences CRC cell growth

**Figure 2:**
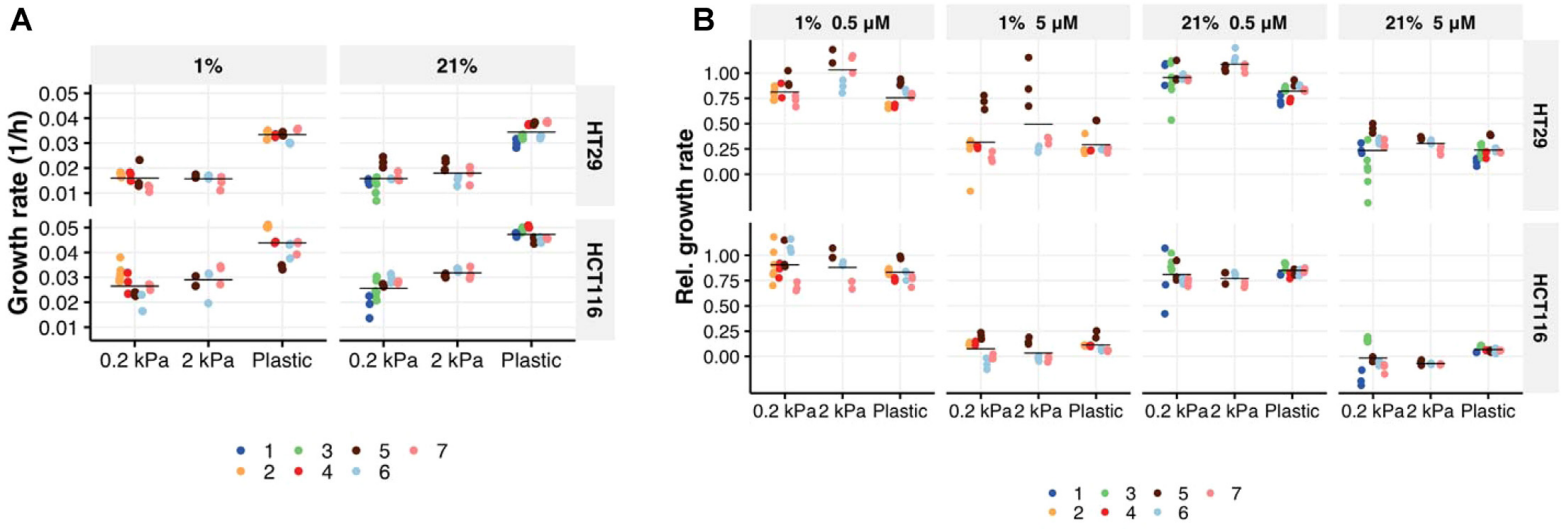
The influence of stiffness on CRC growth and treatment response to oxaliplatin. **A**, HT29-H2BGFP and HCT116-H2BGFP cells were cultured on 0.2 or 2 kPa gel (softwell) or plastic (CellCarrier) plates in 1% or 21% oxygen concentration for 72 hours. Cell counts were measured at several time points by Operetta high-content screening platform, and the growth rate was determined by CellPD. **B**, Relative growth rate of HT29-H2BGFP and HCT116-H2BGFP cells in response to 0.5 or 5 μM oxaliplatin treatment in 1% or 21% oxygen concentration.

A hypoxic tumor environment can lead to the overexpression of ECM proteins by tumor cells, increasing the crosslinking and stiffening of the ECM [38]. The dense fibrotic matrix in solid tumors contributes to local hypoxia through elevating interstitial fluid pressure and disrupting neovascularization [23]. Recent evidence demonstrates that increased stiffness plays a pivotal role in CRC progression and metastasis [25]. Traditional approaches to measure stiffness effects on cancer cell growth involve culturing cells in Matrigel or soft agar; however, their mechanical properties are poorly defined. Here we cultured cells on commercially available collagen-coated polyacrylamide plates (Softwell) with stiffness of 0.2 and 2 kPa (mimicking the stiffness of primary CRC tumors and LMs, respectively) [25]. To longitudinally measure cell growth on the softwells, we generated fluorescently labeled HCT116 and HT29 cells through stable infection with Histone-2B-GFP lentiviruses (HCT116-H2BGFP and HT29-H2BGFP) (Supplementary Fig. S2A). To verify that the transfection did not alter cell behavior, growth rates of HCT116-H2BGFP and HT29-H2BGFP were compared to those of unlabeled cells (Supplementary Fig. S2B). For subsequent investigations into TME-induced cell phenotypes, we used the HCT116-H2BGFP and HT29-H2BGFP cells. Growth rates were evaluated from cells cultured on 0.2 kPa softwell, 2 kPa softwell, and conventional plastic (∼3 GPa) plates using time-series data consisting of live cell counts obtained over a period of 0–72 hours. We found that softer matrices (0.2 and 2 kPa softwell) reduced the growth rate of both cell lines under 1% and 21% oxygen concentrations (Fig. 2A). We observed an increased growth rate of HCT116-H2BGFP cells under 21% oxygen conditions on 2.0 kPa stiffness compared to 0.2 kPa with a posterior mean estimate of 0.006 (95% CI, 0.001–0.011) per hour (Fig. 2A). There was no measured difference (95% CI contains 0) in growth rate between 0.2 and 2 kPa in HT29-H2BGFP cells (Fig. 2A). The sensitivity to oxaliplatin was not altered by stiffness in HCT116 cells; however, a protective effect by softer matrices was observed in HT29-H2BGFP cells under a low dose of oxaliplatin treatment in 21% and 1% oxygen environment, with a mean estimate of 0.21 (95% CI, 0.08–0.33) and 0.16 (95% CI, 0.02–0.29), respectively (Fig. 2B). In addition, compared to 0.2 kPa, we found that 2.0 kPa increased the relative growth rate of low-dose oxaliplatin-treated HT29-H2BGFP cells under 1% oxygen concentrations, with a posterior mean estimate of 0.23 (95% CI, 0.03–0.41) (Fig. 2B).

### CRC cell growth on liver ECM

**Figure 3:**
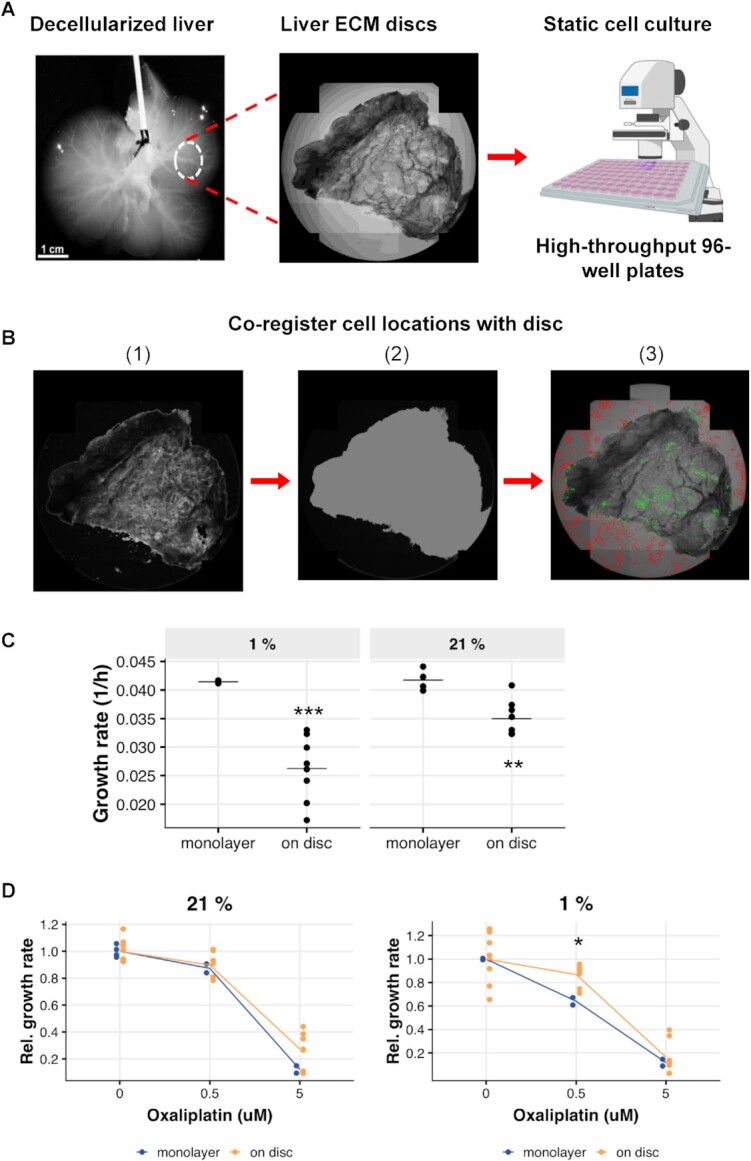
The effect of liver ECM on the growth of CRC cells and treatment response to oxaliplatin. **A**, Liver ECM discs were sectioned from acellular liver and seeded with HCT116-H2BGFP cells. **B**, Snapshots of the disc segmentation process: (1) applying STD filtering and median filtering to the well; (2) applying dilation-reconstruction morphological operations, thresholding and drawing the segmented region over the original image; (3) separating the cells into on-disc (green) and off-disc (red) sets on the basis of the cell location. **C**, HCT116-H2BGFP cells were cultured on liver ECM disc or monolayer under 1% or 21% oxygen concentration for 72 hours. Cell counts were measured at several time points by Operetta HCS platform, and the growth rate was determined by CellPD. Horizontal black line denotes the mean. ^**^P < 0.01 ***P < 0.001 **D**, Relative growth rate of HCT116-H2BGFP cells in response to 0.5 or 5 μM oxaliplatin treatment under 1% or 21% oxygen concentration. *P < 0.05.

The ECM is an essential, yet understudied, component of the TME that physically supports tissues and provides a substrate for cell adhesion and migration, as well as a source of bioactive molecules [18]. To further interrogate the interaction of tumor cells with the metastatic tissue microenvironment, we developed a model of metastatic CRC growth in the liver using acellular liver ECM following our previously published detergent-based perfusion technique [39]. The decellularized liver scaffolds maintain important native ECM components such as collagens, laminin, and fibronectin and retain characteristics of 3D architecture and shape [39]. To quantitatively measure the effect of liver ECM on metastatic CRC growth, we sectioned the acellular livers into circular discs that were then confined in a 96-well plate for screening (Fig. 3A). It has been shown HCT116 is the most efficient line to metastasize to liver in different animal models [35, 40, 41]. Therefore, we chose to seed HCT116-H2BGFP cells on liver ECM discs and imaged longitudinally using our HCS platform. We imported the segmented cell coordinates and disc images (per well) into a MATLAB script to co-register the disc and cell positions and exclude off-disc cells from the calculations (Fig. 3B). This allowed us to separate cells grown on the disc from those that settled on the background well plate. We used CellPD to calculate the growth rate of cells grown on the discs under different oxygen concentrations. Our results showed that the growth kinetics on liver ECM discs are markedly different from those measured on plastic cell culture plates (Fig. 3C). Interestingly, growth on liver ECM makes the HCT116-H2BGFP cells less sensitive to oxaliplatin treatment under hypoxia but not under normoxia (Fig. 3D).

## Discussion

Metastatic growth in distant organ sites is one of the most challenging areas in cancer treatment. Metastasis is a multi-step process, with many studies focusing on molecular changes driving metastatic progression. However, no new gene mutations or amplifications have been clearly linked to metastasis in CRC thus far. The idea that tumor cells “seed” and grow in permissive “soil” was first suggested by Stephen Paget in 1889 [42]. Although Paget's ideas remain relevant today, many of the underlying mechanisms that explain his observations are poorly understood. Key problems in metastasis remain unsolved, including the organ microenvironment's role in seeding, survival, and sustained metastatic growth, and its relations with patient outcome. The TME is heterogenous in nature, but traditional biological assays often only examine a single environmental factor at a time, which is not representative of the biology. Our quantitative high-content imaging approach illuminates the dynamic interactions between cancer cells and treatment response to oxaliplatin under a multiplicity of environmental perturbations, which would be difficult to tune or modulate *in vivo*.

A major determinant of sensitivity to oxaliplatin in CRC cells lines is the p53–p21 pathway [43]. HCT116, a p53 wild-type cell line, is strongly inhibited by oxaliplatin treatment, whereas HT29 cells harboring a p53 mutation are less sensitive to this treatment [44, 45]. Our results showing a reduced growth rate of oxaliplatin-treated HCT116 cells under hypoxia, which is not evident in HT29, support further examination into whether hypoxia alters oxaliplatin-induced p53 activation in CRC cells. It is also interesting to note that HCT116 and HT29 cells are microsatellite instable and microsatellite stable, respectively [44, 45]. It has been shown that moderate hypoxia down-regulates DNA mismatch repair (MMR) genes in a hypoxia-inducible factor (HIF)-dependent manner, while severe hypoxia can lead to transcriptional repression in a HIF-independent manner [16]. Whether hypoxia-induced down-regulation of MMR genes contributes to the differential oxaliplatin IC_50_ observed in our studies deserves further investigation.

Previous research has shown that the increased metastatic potential of HCT116 cells could be due to enhanced ECM adhesion and haptotaxis [41]. Our data suggest that a stiffer LM microenvironment may also contribute to the aggressive phenotype of HCT116 cells. Specifically, we found that HCT116 cells grew faster on the 2 kPa stiffness, a physical environment more similar to LM, than on the 0.2 kPa stiffness, and this was not observed in the less aggressive HT29 cell line. Our results also reveal a liver ECM-driven effect that attenuates oxaliplatin-induced HCT116 growth inhibition under hypoxia conditions, which is not evident in LM-mimicking stiffness (2 kPa softwell). This finding may suggest a stiffness-independent crosstalk between liver ECM and hypoxia signaling.

When we examined the combinatorial impact of oxaliplatin treatment and stiffness, we observed a difference in growth rate in the HT29 cells that was not evident in the HCT116 cells. We found that LM-mimicking stiffness (2 kPa) increased the relative growth rate of low-dose oxaliplatin-treated HT29 cells under 1% oxygen concentration compared to primary tumor-mimicking stiffness (0.2 kPa). Increased matrix stiffness has been shown to increase stemness characteristics and result in oxaliplatin resistance through Akt/mTOR pathway [46]. mTOR can also be affected by hypoxia and mediate additional changes in translation [47]. The multifaced interaction between hypoxia, stiffness, and drug resistance warrants further investigation. An advantage of HCS is the ability to assay complex cellular phenotypes in this multiplexed fashion [32]. Understanding multiple microenvironmental interactions is key to developing therapeutic microenvironmental manipulations in liver and other metastatic sites for controlling metastatic tumor growth.

Our CRC imaging dataset has the potential for extensive reuse in multicellular systems biology. Converting quantitative measurements into cell phenotype parameters with CellPD facilitates data sharing and implementation into dynamical computational models. Several computational models have been developed to investigate the dynamics of more invasive phenotypes driven by oxygen-limited environments, as well as the feedback between multicellular cancer systems and the chemical/biophysical microenvironment [48–51]. The impact of ECM has also been included to simulate tumor-associated angiogenesis [52, 53]. Such simulation investigations have yielded substantial insights on the multicellular dynamics of cancer. However, future advances will necessitate high-quality datasets that can be used to formulate single-cell biological hypotheses (simulated cell “rules”), simulate the emergent multicellular behavior, and validate by comparison with imaging and other data [30].

Recent work, using generic tumor cell phenotypic parameters, showed that relatively simple hypotheses on tumor-stromal mechanobiologic feedbacks can lead to complex emergent behaviors in LM including tumor dormancy [54]. The dataset presented in this article could extend such studies both by providing refined phenotypic parameters and by motivating improved biological hypotheses. In particular, this dataset's measurements on how proliferation varies with tissue stiffness and oxygenation could improve constitutive hypotheses to simulate populations of tumor cells growing in dynamic, heterogeneous conditions. Because we also measured therapeutic response in multiple cell lines in varied TME conditions, those same frameworks could be used to assess evolutionary processes that drive therapeutic resistance in the specific context of the liver parenchyma.

High-throughput quantitative imaging datasets may bridge the gap between traditional biology and computational modeling to enable a systematic investigation of multiple linked microenvironmental factors contributing to CRC metastatic growth and potential therapeutic strategies. The cell phenotype parameters generated from our HCS platform will help build experimentally driven computational models of metastatic colon cancer cell growth as a function of microenvironment conditions in the liver parenchyma. We can then use these models of metastatic tumor growth to probe the relationships between growth dynamics and heterogeneous microenvironments to facilitate a deeper understanding of complex metastatic processes, and to develop new hypotheses and possible therapeutic interventions. We also envision that multifactorial datasets (including this one) will serve as gold standard data to help drive refinements in dynamic simulation model calibration and validation protocols.

## Methods

### Cell culture and reagents

The human colorectal cell lines HCT116 and HT29 were acquired from ATCC and cultured in McCoy's 5A medium supplemented with 10% fetal bovine serum (Gemini, West Sacramento, CA, USA) and 1% penicillin/streptomycin (Gemini, West Sacramento, CA, USA). Caco2 cells were acquired from ATCC and maintained in Eagle's Minimum Essential Medium supplemented with 10% fetal bovine serum and 1% penicillin/streptomycin. For live cell imaging, HCT116-H2BGFP and HT29-H2BGFP were created by transducing HCT116 and HT29 with LentiBrite Histone H2B-GFP lentivirus (Millipore #17–10229, Burlington, MA, USA). A positive green fluorescent protein (GFP) cell population was selected by a fluorescence-activated cell sorter. Cell lines were authenticated by a professional authentication service (University of Arizona Genetic Core) and routinely tested for mycoplasma contamination using MycoAlert (Lonza No. LT07–518, Basel, Switzerland). Hypoxia experiments were carried out in a hypoxia workstation (Biospherix, Parish, NY, USA) with separate chambers that allow for precise control over oxygen culture conditions (0.1–1% O_2_).

### Liver ECM disc preparation

Following our published protocol, livers from ferrets age 5–6 weeks were harvested and decellularized using a detergent of deionized water, 1% triton X-100, and 0.1% ammonium hydroxide for 3 days. The spatial arrangement of collagens I, III, and IV, laminin, and fibronectin is similar to that in fresh human liver tissue [39]. Decellularized livers were embedded in OCT compound for frozen sectioning. The tissue was then sectioned into circular discs with a diameter of 6 mm. The discs were confined in 96-well CellCarrier plates with PBS to prevent tissue dehydration. Prior to CRC cell seeding, discs were washed 3 more times with PBS and then pre-conditioned with culture medium at 37°C for 60 minutes.

### Image acquisition and analysis

End point growth rate experiments of HCT116, HT29, and Caco2 were carried out in 96-well CellCarrier plates (PerkinElmer No. 6005558, Waltham, MA, USA) at an initial cell seeding of 1,500, 4,000, and 2,000 cells per well, respectively. One day after seeding, cells were treated with the indicated dilutions of oxaliplatin (Selleck Chemicals No. S1224, Houston, TX, USA). At the stated time points, images were acquired on an Operetta HCS System (PerkinElmer No. HH12000000) equipped with environmental controls (37°C, 5% CO_2_). Thirty minutes prior to imaging, cells were stained with 5 μg/mL of Hoechst 33342 (Invitrogen No. H21492, Carlsbad, CA, USA) and 5 μg/mL of propidium iodine (Invitrogen No. P1304MP, Carlsbad, CA, USA) to determine live or dead cells, respectively. For live cell experiments, cells were seeded on 0.2 or 2 kPa softwell (Matrigen) or CellCarrier plates in the presence or absence of liver ECM disc. Images were taken on the Operetta HCS in confocal mode using the *z*-stack function. For all experiments, image analysis was performed using the Harmony 3.5.2 software (PerkinElmer No. HH17000001, Waltham, MA, USA). Cells were identified and segmented at the nuclear level to determine live and dead cell counts over time as described previously [32].

### Determination of IC_50_

The IC_50_ value was determined for each experiment by estimating the oxaliplatin concentration at which the growth rate was 50% of the untreated value via linear interpolation on a log-concentration scale.

### Register cells on liver ECM discs

A 2-step process was used to separate cells seeded on the disc and on the background well plate. We first segmented the image of the well to on- and off-disc regions and then co-registered the cell locations with the disc region.

### Segmenting the disc

Given that the local variance at the off-disc region is lower compared to the disc regions, we used a standard deviation (STD) filter followed by a median filter to find the main structure of the disc and then applied a series of morphological operations to include the small details and trim the noisy non-disc regions close to the borders. We also imaged empty wells to make a light profile for the images and then compared this profile with the images of the wells to add some candidate on-disc pixels before applying morphological operations. We ran the disc segmentation by sweeping over the parameters that control the segmentation and manually chose the best segmentation. The 3 main parameters used were (i) the kernel size for the STD filtering, (ii) the threshold for marking a pixel as a candidate on-disc pixel, and (iii) the size of structural elements used for morphological operations.

### Co-registering the cell locations with disc

Cell segmentation and the cell's center coordinates were acquired from the Harmony 3.5.2 software. The center of the cell was overlayed with the segmented mask region. By iterating over all the cells, we separated the cells on the basis of location on or off the disc.

### Statistical Analysis

Figure 1B. Increasing or decreasing growth rate across O_2_ levels was tested using a 2-sided sign test across all same-experiment increasing O_2_ levels. This nonparametric procedure is insensitive to cross-experiment measurement variation, and uses only the order of O_2_ levels and not their specific values. The tests were sufficiently powered to detect instances where all data trended in a single direction at a significance level of *P* = 0.05 (Caco2: 6 comparisons; HT29: 7 comparisons; HCT116: 8 comparisons). This criterion was met only for HCT116. Sign tests were performed in R using the SIGN.test function in the BSDA package (v1.2.0).

Figure 1D. We tested for IC_50_ differences between hypoxia (1% or 0.1% O_2_) and normoxia (21% O_2_) using posterior estimates from an empirical Bayesian model (brms package v2.13.5 under R v4.0.2). The model included effects for each hypoxia comparison to normoxia for each cell line. Weakly informative priors were used both for IC_50_ differences (Gaussian) and noise level (Cauchy), each scaled loosely to the data. Reported credible intervals are symmetric 95% intervals of the IC_50_ difference posterior distributions.

Figure 2. We quantified growth rate differences (absolute and relative) from posterior distributions of empirical Bayesian models. Models included categorical population (fixed) effects for cell type (HCT116, HT29) and plate type (Plastic, Softwell 0.2 kPa, Softwell 2 kPa), and a group (random) effect for experiment date (consisting of 1 plate for each). Residual Gaussian errors were grouped by plate type, each having its own variance estimate. Priors on growth rates (absolute and relative), interplate variance, and residual variance were all Cauchy distributed, with zero mean and width order-of-magnitude empirically derived from the data. Estimates of growth rates and their differences are reported as posterior mean and 95% credible (posterior) intervals.

Figure 3. Reported *P*-values are computed from 2-sided Welch *t*-tests.

## Data Availability

The datasets underlying this article are available in the*GigaScience* GigaDB database [55].

## Abbreviations

95% CI: 95% credible interval; CRC: colorectal cancer; ECM: extracellular matrix; GFP: green fluorescent protein; HCS: high-content screening; IC_50_: half maximal inhibitory concentration; LM: liver metastasis; MMR: mismatch repair; OCT: optimal cutting temperature; PBS: phosphate-buffered saline; STD: standard deviation; TME: tumor microenvironment.

## Funding

This work was supported by a National Institutes of Health/National Cancer Institute R01 Provocative Questions (PQ) Grant, CA180149 (awarded to D.B.A., A.A., and S.S.). Additional support was provided by the University of Southern California Medical Faculty Women's Association (awarded to S.M.M.).

## Competing Interests

The authors declare that they have no competing interests.

## Authors' Contributions

C.T.C. and R.L. conducted experiments and analyzed data. A.G. and P.M. wrote MATLAB script to co-register cell locations with disc. E.F.J. and P.M. wrote CellPD to calculate the cell growth rate. D.R. performed the statistical analyses. D.V., M.B., S.S., and A.A. generated liver ECM discs. C.T.C., D.B.A., D.R., P.M., and S.M.M. wrote the manuscript and conceptualized the framework for this research. All authors helped edit the manuscript.

## Supplementary Material

giab026_GIGA-D-20-00103_Original_Submission

giab026_GIGA-D-20-00103_Revision_1

giab026_Response_to_Reviewer_Comments_Original_Submission

giab026_Reviewer_1_Report_Original_Submission Chris Armit -- 4/17/2020 Reviewed

giab026_Reviewer_2_Report_Original_Submission Ramya Sivakumar -- 6/16/2020 Reviewed

giab026_Reviewer_3_Report_Original_Submission Jessica Bauer -- 6/22/2020 Reviewed

giab026_Reviewer_3_Report_Revision_1 Jessica Bauer -- 12/10/2020 Reviewed

giab026_Supplemental_File
