# Supplementary material for: High-throughput microscopy reveals the impact of multifactorial environmental perturbations on colorectal cancer cell growth
Source: Gigascience. 2021 Apr 19;10(4):giab026. doi: 10.1093/gigascience/giab026 (PMC8054261; doi:10.1093/gigascience/giab026)
Supplement: giab026_GIGA-D-20-00103_Original_Submission [file giab026_giga-d-20-00103_original_submission.pdf]

GigaScience
High throughput microscopy reveals the impact of multifactorial environmental perturbations on colorectal cancer cell growth
--Manuscript Draft--

**Manuscript Number:**	GIGA-D-20-00103
**Full Title:**	High throughput microscopy reveals the impact of multifactorial environmental perturbations on colorectal cancer cell growth
**Article Type:**	Research
**Funding Information:**	National Cancer Institute (R01CA180149) — Dr. David B. Agus
**Abstract:**	Background: Colorectal cancer (CRC) mortality is principally due to metastatic disease, with the most frequent organ of metastasis being liver. Biochemical and mechanical factors residing in the tumor microenvironment (TME) are considered to play a pivotal role in metastatic growth and response to therapy. However, it is difficult to study the TME systematically due to a lack of fully controlled model systems that can be investigated in rigorous detail.<br><br>Results: We present a quantitative imaging dataset of CRC cell growth dynamics influenced by in vivo -mimicking conditions. They consist of tumor cells grown in various biochemical and biomechanical microenvironmental contexts. These contexts include varying oxygen and drug concentrations, and growth on conventional stiff plastic, softer matrices, and bioengineered acellular liver extracellular matrix (ECM). Growth rate analyses under these conditions were performed via the cell phenotype digitizer (CellPD).<br><br>Conclusions: Our data indicate the growth of highly aggressive HCT116 cells is affected by oxygen, substrate stiffness, and liver ECM. In addition, hypoxia has a protective effect against oxaliplatin-induced cytotoxicity on plastic and liver ECM. This expansive data set of CRC cell growth measurements under in situ relevant environmental perturbations provides insights into critical TME features contributing to metastatic seeding and tumor growth. Such insights are essential to modeling and understanding the multicellular tumor-stroma dynamics that contribute to metastatic colonization. It also establishes a benchmark data set for training and testing data-driven computational models of cancer and other multicellular systems.
**Corresponding Author:**	Shannon Mumenthaler<br>University of Southern California Health Sciences Center<br>Los Angeles, California UNITED STATES
**Corresponding Author Secondary Information:**	
**Corresponding Author's Institution:**	University of Southern California Health Sciences Center
**Corresponding Author's Secondary Institution:**	
**First Author:**	Chun-Te Chiang, Ph.D.
**First Author Secondary Information:**	
**Order of Authors:**	Chun-Te Chiang, Ph.D.
	Roy Lau
	Ahmadreza Ghaffarizadeh, Ph.D.
	Matthew Brovold
	Dipen Vyas, Ph.D.
	Edwin F Juárez, Ph.D.
	Anthony Atala, M.D.
	David B. Agus, M.D.

Powered by Editorial Manager® and ProduXion Manager® from Aries Systems Corporation

	Shay Soker, Ph.D.
	Paul Macklin, Ph.D.
	Daniel Ruderman, Ph.D.
	Shannon M Mumenthaler, PhD
**Order of Authors Secondary Information:**	
**Additional Information:**	
**Question**	**Response**
Are you submitting this manuscript to a special series or article collection?	No
**Experimental design and statistics**<br><br>Full details of the experimental design and statistical methods used should be given in the Methods section, as detailed in our Minimum Standards Reporting Checklist. Information essential to interpreting the data presented should be made available in the figure legends.<br><br>Have you included all the information requested in your manuscript?	Yes
**Resources**<br><br>A description of all resources used, including antibodies, cell lines, animals and software tools, with enough information to allow them to be uniquely identified, should be included in the Methods section. Authors are strongly encouraged to cite Research Resource Identifiers (RRIDs) for antibodies, model organisms and tools, where possible.<br><br>Have you included the information requested as detailed in our Minimum Standards Reporting Checklist?	Yes
**Availability of data and materials**<br><br>All datasets and code on which the conclusions of the paper rely must be either included in your submission or deposited in publicly available repositories (where available and ethically appropriate), referencing such data using a unique identifier in the references and in the "Availability of Data and Materials" section of your manuscript.<br><br>Have you have met the above requirement as detailed in our Minimum Standards Reporting Checklist?	Yes

Powered by Editorial Manager® and ProduXion Manager® from Aries Systems Corporation

Manuscript

Click here to download Manuscript Manuscript_Giga Science_v8.docx

Title: High throughput microscopy reveals the impact of multifactorial environmental perturbations on colorectal cancer cell growth

Author Affiliations: Chun-Te Chiang[1*], Roy Lau[1*], Ahmadreza Ghaffarizadeh[1], Matthew Brovold[2], Dipen Vyas[2], Edwin F. Juárez[1], Anthony Atala[2], David B. Agus[1], Shay Soker[2], Paul Macklin[1,3], Daniel Ruderman[1ǂ], Shannon M. Mumenthaler[1ǂ]

[1] Lawrence J. Ellison Institute for Transformative Medicine, University of Southern California, Los Angeles, California, USA
[2] Wake Forest Institute for Regenerative Medicine, Winston Salem, North Carolina, USA
[3] Intelligent Systems Engineering, Indiana University, Bloomington, IN, USA
[*] These authors contributed equally to this work
[ǂ] Co-corresponding authors

Corresponding Authors

D.R.: Department of Medicine, University of Southern California, 2250 Alcazar Street, CSC 240, Los Angeles, CA 90033. Phone: (323) 442-2839. E-mail: ruderman@usc.edu

S.M.: Department of Medicine, University of Southern California, 2250 Alcazar Street, CSC 242, Los Angeles, CA 90033. Phone: (323) 442-2529. E-mail: smumenth@usc.edu

Abstract

Background: Colorectal cancer (CRC) mortality is principally due to metastatic disease, with the most frequent organ of metastasis being liver. Biochemical and mechanical factors residing in the tumor microenvironment (TME) are considered to play a pivotal role in metastatic growth and response to therapy. However, it is difficult to study the TME systematically due to a lack of fully controlled model systems that can be investigated in rigorous detail.

Results: We present a quantitative imaging dataset of CRC cell growth dynamics influenced by *in vivo*-mimicking conditions. They consist of tumor cells grown in various biochemical and biomechanical microenvironmental contexts. These contexts include varying oxygen and drug concentrations, and growth on conventional stiff plastic, softer matrices, and bioengineered acellular liver extracellular matrix (ECM). Growth rate analyses under these conditions were performed via the cell phenotype digitizer (CellPD).

Conclusions: Our data indicate the growth of highly aggressive HCT116 cells is affected by oxygen, substrate stiffness, and liver ECM. In addition, hypoxia has a protective effect against oxaliplatin-induced cytotoxicity on plastic and liver ECM. This expansive data set of CRC cell growth measurements under *in situ* relevant environmental perturbations provides insights into critical TME features contributing to metastatic seeding and tumor growth. Such insights are essential to modeling and understanding the multicellular tumor-stroma dynamics that contribute to metastatic colonization. It also establishes a benchmark data set for training and testing data-driven computational models of cancer and other multicellular systems.

Keywords: Colorectal Cancer, High Content Imaging, Liver Metastasis, Tumor Microenvironment

Background

Colorectal cancer (CRC) is the third most deadly cancer in both man and woman in the United States [1]. Current treatment strategies include FOLFOX (5-FU, leucovorin and oxaliplatin), FOLFIRI (5-FU, leucovorin and irinotecan) or XELOX (oxaliplatin and capecitabine) with or without molecular targeted drugs [2]. The 5-year survival rate for CRC is 90% if the cancer is diagnosed locally. However, once the disease has spread to distant sites, this rate drops dramatically to around 10% [3]. Emerging data, spanning from clinical to laboratory research, highlights that metastatic disease cannot be explained solely by the genetics of the cancer cells; instead, bidirectional interactions with the surrounding microenvironment play a pivotal role in tumor progression [4-7]. Devising innovative ways to treat CRC metastasis needs to address not only the genetic heterogeneity of the tumor but also its dynamic microenvironment.

Liver is the most common organ of distant metastasis. More than 50% of CRC patients with advanced disease develop liver metastases [8]. Many such metastases are discovered months or years after initial seeding. Consequently, most patients require treatment for established liver tumor foci. It has been postulated that the high rate of liver metastases is a result of anatomical considerations, with the portal vein draining directly from the colon and upper rectum to the liver [9]. However, increasing evidence has demonstrated that the liver microenvironment is vital in influencing CRC metastasis [10-12]. Low oxygen levels (hypoxia) and altered extracellular matrix (ECM) have been considered key features of the tumor microenvironment (TME) [13]. Poor vascularization in the tumor mass can lead to an oxygen-limited environment and initiate more aggressive phenotypes [14]. Cellular interactions with the surrounding ECM can also regulate a vast range of biological outcomes including disease progression and drug resistance [15]. Different tissues are known to have distinct ECM molecular compositions and architectures. The complexity of the ECM is not merely biochemical: associated mechanical properties, like stiffness, can also greatly impact cell proliferation and motility [16]. Moreover, adhesive tumor cell interactions with liver cells—particularly endothelial cells and hepatocytes in the sinusoids—have recently been shown to impact metastatic progression [17] as well chemical communication with stellate, Kupffer, and inflammatory cells [18].

The TME is a highly complex system with many factors working in concert. However, traditional biological assays often only examine a single environmental factor in a qualitative way, and thereby lack the ability to recapitulate essential features of metastatic growth. Multicellular computational modeling can provide novel insights to link cancer progression to heterogeneous TME conditions and the dynamical interactions between tumor cells and the resident cell ecosystem [19, 20]. However, predicting the impact of liver microenvironmental manipulations on CRC behavior requires high-quality benchmark datasets to fit model parameters and drive the development of computational models [21], particularly systems that can independently and separately study the role of metastatic tumor cell interactions with hypoxia, the ECM, and resident liver cells. High content screening (HCS), the application of automated microscopy and image analysis, has been widely used in cell biology and drug development to screen drug compounds for safety and toxicity on human cells *in vitro* [22]. This platform is also well suited for exploring the impact of multiple TME parameters, either individually or simultaneously, on cell behavior [23, 24]. Here we extend the use of our previously established imaging workflow to study the biophysical and biochemical impact of the organ context, specifically the combination of oxygen, stiffness, and liver ECM, on CRC cell growth and response to therapy. After isolating the impact of hypoxia and ECM biomechanics on CRC cell seeding, we can control for these factors and continue high-throughput investigations that isolate and characterize adhesive and other multicellular interactions during metastatic colonization.

Results

CRC growth rates under different oxygen tensions

Tumor hypoxia, or oxygen deprivation, has been shown to decrease proliferation and limit cells' responsiveness to therapeutic agents [25]. To investigate the impact of hypoxia on CRC growth, we examined three human CRC cell lines with different aggressiveness (Caco2; HT29; HCT116) grown under various oxygen concentrations (normoxic-21%; hypoxic-1% and 0.1%). HCT116 was derived from a poorly differentiated human colon adenocarcinoma known to develop hepatic metastases efficiently in immunodeficient mice. HT29 also has metastatic capabilities but less efficient compared with HCT116. In contrast, Caco2 shows no ability to metastasize [26]. We used the Operetta high content imaging system and Harmony software (Version 3.5.2) to measure cell counts of CRC cell lines grown in the respective oxygen conditions (Fig. 1A and S1). To extract growth rates from sequential cell count data, we utilized CellPD (cell phenotype digitizer) (Version 1.0.1), a previously developed open source Python code which leverages the Levenberg-Marquardt algorithm to perform nonlinear least squares minimization between simulated and experimental cell counts [27]. We observed a trend toward reduced growth rates under the hypoxic conditions in the most aggressive cell line HCT116 (p=0.008), which is not present in the less aggressive cell lines, HT29 and Caco2 (Fig. 1B).

The impact of hypoxia on drug response

Oxaliplatin is a standard CRC chemotherapy agent used clinically. To examine the effect of oxygen on cancer treatment response, we measured CRC growth effects of oxaliplatin under different oxygen concentrations (various concentrations of oxygen – 20, 1, 0.1% and oxaliplatin treatment – 0, 0.062, 0.185, 0.555, 1.667, 5 μM). We found the least aggressive cell line, Caco2, to be most sensitive to oxaliplatin treatment compared to HT29 and HCT116 across all oxygen concentrations (Fig. 1C). Interestingly, the IC_{50} of oxaliplatin was not significantly altered under hypoxia in both Caco2 and HT29 cells. However, there is a credible difference in the IC_{50} of oxaliplatin between 0.1 and 21% oxygen with a mean estimate of 1.73-fold increase (95% credible interval: 1.02 to 3.01) in HCT116 cells (Fig. 1D).

Matrix stiffness environment influences CRC cell growth

Recent evidence shows that mechanical forces regulate tumorigenesis and metastasis [16, 28], illustrating the importance of stiffness as a parameter to consider in TME. Traditional approaches to measure stiffness effects on cancer cell growth involves culturing cells in Matrigel or soft agar; however, the mechanical properties are poorly defined. Here we cultured cells on commercially available collagen-coated polyacrylamide plates (Softwell) with a stiffness of 0.2kPa. To longitudinally measure cell growth on the softer gel, we generated fluorescently labeled HCT116 and HT29 cells through stable infection with Histone-2B-GFP lentiviruses (HCT116-H2BGFP and HT29-H2BGFP) (Fig. S2A). To verify the transfection did not alter cell behavior, growth rates of HCT116-H2BGFP and HT29-H2BGFP were compared to unlabeled cells (Fig. S2B). For subsequent investigations into TME-induced cell phenotypes we used the HCT116-H2BGFP and HT29-H2BGFP cells. Growth rates were evaluated from cells cultured on gel (0.2kPa) versus conventional plastic (~3GPa) plates using time series data consisting of live cell counts obtained over a period of 0-72 hours. We found softer matrices reduced the growth rate of both cell lines under 1 and 21% oxygen concentrations (Fig. 2A). The sensitivity to oxaliplatin was not significantly altered by stiffness except a protective effect by softer matrices was observed in HT29-H2BGFP cells under a low dose of oxaliplatin treatment in 21% oxygen environment (Fig.2B).

CRC cell growth on liver ECM

The ECM is an essential, yet understudied, component of the tumor microenvironment that physically supports tissues and provides a substrate for cell adhesion and migration, as well as a source of bioactive molecules [15]. To further interrogate the interaction of tumor cells with the metastatic tissue microenvironment, we developed a model of metastatic CRC growth in the liver using acellular liver ECM following our previously published detergent-based perfusion technique [29]. The decellularized liver scaffolds maintain important native ECM components such as collagens, laminin and fibronectin, and retain characteristic of 3D architecture and shape [29]. To quantitatively measure the effect of liver ECM on metastatic CRC growth, we sectioned the acellular livers into circular discs that were then confined in a 96-well plate for screening (Fig. 3A). HCT116-H2BGFP cells were seeded on liver ECM discs and imaged longitudinally using our HCS platform. We imported the segmented cell coordinates and disc images (per well) into a MATLAB script to co-register the disc and cell positions and exclude off-disc cells from the calculations (Fig. 3B). This allowed us to separate cells grown on the disc with those that settled on the background well plate. We used CellPD to calculate the growth rate of cells grown on the discs under different oxygen concentrations. Our results showed that the growth kinetics on liver ECM discs are markedly different from those measured on plastic cell culture plates (Fig. 3C). Interestingly, growth on liver ECM makes the HCT116-H2BGFP cells less sensitive to oxaliplatin treatment under hypoxia, but not under normoxia (Fig. 3D).

Discussion

Metastatic growth in distant organ sites is one of the most challenging areas in cancer treatment. Metastasis is a multi-step process with many studies focusing on molecular changes driving metastatic progression. However, no new gene mutations or amplifications have been clearly linked to metastasis in CRC thus far. The idea that tumor cells “seed” and grow in permissive “soil” was first suggested by Stephen Paget in 1889 [30]. Although Paget’s ideas remain relevant today, many of the underlying mechanisms that explain his observations are poorly understood. Key problems in metastasis remain unsolved, including the organ microenvironment’s role in seeding, survival, and sustained metastatic growth, and its relations with patient outcome. The TME is heterogenous in nature, but traditional biological assays often only examine a single environmental factor at a time, which is not representative of the biology. Our quantitative high-content imaging approach illuminates the dynamic interactions between cancer cells and treatment response to oxaliplatin under a multiplicity of environmental perturbations, which would be difficult to tune or modulate *in vivo*. Previous studies have demonstrated that hypoxia results in resistance to oxaliplatin treatment in the highly metastatic HCT116 cells through induction of HIF-1α expression [31, 32]. Our results are not only consistent with these previous findings in monolayer, but also reveal a liver ECM-driven effect that attenuates oxaliplatin-induced growth inhibition under hypoxia condition. These results suggest a potential crosstalk between liver ECM and hypoxia signaling. Understanding multiple microenvironmental interactions is key to developing therapeutic microenvironmental manipulations in liver and other metastatic sites for controlling metastatic tumor growth.

Our CRC imaging dataset has the potential for extensive reuse in multicellular systems biology. Converting quantitative measurements into cell phenotype parameters with CellPD facilitates data sharing and implementation into computational models. Several computational models have been developed to investigate the dynamics of more invasive phenotypes driven by oxygen-limited environments, as well as the feedback between multicellular cancer systems and the chemical / biophysical microenvironment [33-36]. The impact of ECM has also been included to simulate tumor-associated angiogenesis [37, 38]. Such simulation investigations have yielded substantial insights on the multicellular dynamics of cancer. However, future advances will require high-

quality data sets that can be used to formulate single-cell biological hypotheses (simulated cell "rules"), simulate the emergent multicellular behavior, and validate by comparison with imaging and other data [21].

High-throughput quantitative imaging datasets may bridge the gap between traditional biology and computational modeling to enable a systematic investigation of multiple linked microenvironmental factors contributing to CRC metastatic growth and potential therapeutic strategies. The cell phenotype parameters generated from our HCS platform will help build experimentally-driven computational models of metastatic colon cancer cell growth as a function of microenvironment conditions in the liver parenchyma. We can then use these models of metastatic tumor growth to probe the relationships between growth dynamics and heterogeneous microenvironments to facilitate a deeper understanding of complex metastatic processes, and to develop new hypotheses and possible therapeutic interventions. We also envision that multifactorial datasets (including this one) will serve as gold standard data to help drive refinements in simulation model calibration and validation protocols.

Methods

Cell culture and reagents

The human colorectal cell lines HCT116 and HT29 were acquired from ATCC and cultured in McCoy's 5A medium supplemented with 10% fetal bovine serum (Gemini) and 1% penicillin/streptomycin (Gemini). Caco2 cells were acquired from ATCC and maintained in EMEM supplemented with 20% fetal bovine serum and 1% penicillin/streptomycin. For live cell imaging, HCT116-H2BGFP and HT29-H2BGFP were created by transducing HCT116 and HT29 with LentiBrite Histone H2B-GFP lentivirus (Millipore #17-10229). A positive GFP cell population was selected by a fluorescence-activated cell sorter (FACS). Cell lines were authenticated by a professional authentication service (University of Arizona Genetic Core) and routinely tested for mycoplasma contamination using MycoAlert (Lonza #LT07-518). Hypoxia experiments were carried out in a hypoxia workstation (Biospherix) with separate chambers that allow for precise control over oxygen culture conditions (0.1% - 1% O2).

Liver ECM disc preparation

Following our published protocol, livers from ferrets age 5-6 weeks were harvested and decellularized using a detergent of deionized water, 1% triton X-100 and 0.1% ammonium hydroxide for three days. The spatial arrangement of collagens I, III and IV, laminin and fibronectin is similar to fresh human liver tissue [29]. Decellularized livers were embedded in OCT for frozen sectioning. The tissue was then sectioned into circular discs with a diameter of 6 mm. The discs were confined in 96 well CellCarrier plates with PBS to prevent tissue dehydration. Prior to CRC cell seeding, discs were washed 3 more times with PBS and then pre-conditioned with culture medium at 37°C for 60 minutes.

Image acquisition and analysis

Endpoint growth rate experiments of HCT116, HT29 and Caco2 were carried out in 96 well CellCarrier plates (PerkinElmer #6005558) at an initial cell seeding of 1500, 4000, and 2000 cells per well, respectively. One day after seeding, cells were treated with the indicated dilutions of oxaliplatin (Selleck Chemicals #S1224). At the stated time points, images were acquired on an Operetta High Content Screening (HCS) System (PerkinElmer #HH12000000) equipped with environmental controls (37°C, 5% CO_2). 30 minutes prior to imaging, cells were stained with 5 μg/ml of Hoechst 33342 (Invitrogen #H21492) and 5 μg/ml of propidium iodine (Invitrogen #P1304MP) to determine live or dead cells, respectively. For live cell experiments, cells were

seeded on 0.2kPa softwell (Matrigen) or CellCarrier plates in the presence or absence of liver ECM disc. Images were taken on the Operetta HCS in confocal mode using the z-stack function. For all experiments, image analysis was performed using the Harmony 3.5.2 software (PerkinElmer #HH17000001). Cells were identified and segmented at the nuclear level to determine live and dead cell counts over time as described previously [23].

Determination of IC_{50}

The IC_{50} value was determined for each experiment by estimating the oxaliplatin concentration at which the growth rate was 50% of the untreated value via linear interpolation on a log-concentration scale.

Register cells on liver ECM discs

A two-step process was used to separate cells seeded on the disc and on the background well plate. We first segmented the image of the well to on- and off-disc regions and then co-registered the cell locations with the disc region.

<u>Segmenting the disc</u>

Given the local variance at the off-disc region is lower compared to the disc regions, we employed a standard deviation (STD) filter followed by a median filter to find the main structure of the disc and then applied a series of morphological operations to include the small details and trim the noisy non-disc regions close to the borders. We also imaged empty wells to make a light profile for the images and then compared this profile with the images of the wells to add some candidate on-disc pixels before applying morphological operations. We ran the disc segmentation by sweeping over the parameters that control the segmentation and manually chose the best segmentation. The three main parameters used were 1) the kernel size for the STD filtering, 2) the threshold for marking a pixel as a candidate on-disc pixel, and 3) the size of structural elements used for morphological operations.

<u>Co-registering the cell locations with disc</u>

Cell segmentation and the cell's center coordinates were acquired from the Harmony 3.5.2 software. The center of the cell was overlayed with the segmented mask region. By iterating over all the cells, we separated the cells based on a location on or off the disc.

Statistical Analysis

<u>*Figure 1.*</u> 1B: Increasing or decreasing growth rate across O_2 levels was tested using a two-sided sign test across all same-experiment increasing O_2 levels. This nonparametric procedure is insensitive to cross-experiment measurement variation, and employs only the order of O_2 levels, and not their specific values. The tests were sufficiently powered to detect instances where all data trended in a single direction at a significance level of p=0.05 (Caco2: 6 comparisons, HT29: 7 comparisons, HCT116: 8 comparisons). This criterion was met only for HCT116. Sign tests were performed in *R* using the *SIGN.test* function in the *BSDA* package (v1.2.0). 1D: We tested for IC_{50} differences between hypoxia (1% or 0.1% O_2) and normoxia (21% O_2) using posterior estimates from a Bayesian model (*R brms* package v2.10.0). The model included effects for each hypoxia comparison to normoxia for each cell line. Weakly informative priors were used both for IC_{50} differences (Gaussian) and noise level (Cauchy), each scaled loosely to the data. Reported credible intervals are symmetric 95% intervals of the IC_{50} difference posterior distributions.
<u>*Figure 2.*</u> We quantified growth rate differences (absolute and relative) using a Bayesian model. It included population (fixed) effects for cell type and plate type (CellCarrier, Softwell), and a group (random) effect for experiment date. Residual Gaussian errors were grouped by plate type, each

with its own variance estimate. Results are reported as posterior mean and posterior 95% intervals.
Figure 3. Reported p-values are computed from two-sided Welch's t-tests.

Competing interests

The authors declare no competing interests.

Author contributions

C.T.C. and R.L. conducted experiments and analyzed data. A.G. and P.M. wrote MATLAB script to co-register cell locations with disc. E.F.J. and P.M. wrote CellPD to calculate cell growth rate. D.R. performed the statistical analyses. D.V., M.B., S.S., and A.A. generated liver ECM discs. C.T.C., D.A., D.R. and S.M.M. wrote the manuscript and conceptualized the framework for this research. All authors helped edit the manuscript.

Acknowledgement

This work was funded by a National Cancer Institute Grant R01 CA180149. Additional support was provided by the USC Medical Faculty Women's Association (awarded to S.M.M.). We thank A. Skardal (Ohio State University) and R. Sun for technical support and N. Matasci and T. Gerhart for script maintenance. We would also like to express our deepest gratitude to our philanthropic supporters: the Stephenson family, Emmet, Toni and Tessa, for their donation of the Operetta HCS platform, which was instrumental in carrying out this work.

Figure titles and legends

Fig. 1. The impact of oxygen on CRC growth and treatment response to oxaliplatin. a Caco2, HT29, and HCT116 cells were cultured in 0.1%, 1% or 21% oxygen concentration. Cell counts were measured at several time points using Operetta high-content screening platform. **b** Growth rate of Caco2, HT29, and HCT116 cells in 0.1%, 1% or 21% oxygen concentration was determined by CellPD. A two-sided sign test was used to detect instances where all data trended in a single direction. **c** Relative growth rate of Caco2, HT29, and HCT116 cells treated with 0.167, 0.5, 1.67, 5 μM oxaliplatin. **d** Oxaliplatin IC_{50} changes in 0.1, 1 or 21% oxygen environment.

Fig. 2. The influence of stiffness on CRC growth and treatment response to oxaliplatin. a HT29-H2BGFP and HCT116-H2BGFP cells were cultured on gel (softwell) or plastic (CellCarrier) plate in 1% or 21% oxygen concentration for 72 hours. Cell counts were measured at several time points by Operetta high-content screening platform, and the growth rate was determined by CellPD. **b** Relative growth rate of HT29-H2BGFP and HCT116-H2BGFP cells in response to 0.5 or 5 μM oxaliplatin treatment in 1% or 21% oxygen concentration.

Fig.3. The effect of liver ECM on the growth of CRC cells and treatment response to oxaliplatin. a Liver ECM discs were sectioned from acellular liver and seeded with HCT116-H2BGFP cells. **b** Snapshots of the disc segmentation process: 1) applying STD filtering and median filtering to the well; 2) applying dilation-reconstruction morphological operations, thresholding and drawing the segmented region over the original image; 3) separating the cells to on-disc and off-disc sets based on the cell location. The on-disc cells were colored green and off-disc cells were red. **c** HCT116-H2BGFP cells were cultured on liver ECM disc or monolayer under 1% or 21% oxygen concentration for 72 hours. Cell counts were measured at several time points by Operetta HCS platform, and the growth rate was determined by CellPD. **d** Relative growth rate of HCT116-H2BGFP cells in response to 0.5 or 5 μM oxaliplatin treatment under 1% or 21 % oxygen concentration. ***$P < 0.001$, **$P < 0.01$, *$P < 0.05$

References

1. Siegel RL, Miller KD and Jemal A. Cancer statistics, 2019. CA Cancer J Clin. 2019;69 1:7-34. doi:10.3322/caac.21551.
2. Sanchez-Gundin J, Fernandez-Carballido AM, Martinez-Valdivieso L, Barreda-Hernandez D and Torres-Suarez AI. New Trends in the Therapeutic Approach to Metastatic Colorectal Cancer. Int J Med Sci. 2018;15 7:659-65. doi:10.7150/ijms.24453.
3. Lee RM, Cardona K and Russell MC. Historical perspective: Two decades of progress in treating metastatic colorectal cancer. J Surg Oncol. 2019;119 5:549-63. doi:10.1002/jso.25431.
4. Willyard C. Cancer therapy: an evolved approach. Nature. 2016;532 7598:166-8. doi:10.1038/532166a.
5. Tan Q, Saggar JK, Yu M, Wang M and Tannock IF. Mechanisms of Drug Resistance Related to the Microenvironment of Solid Tumors and Possible Strategies to Inhibit Them. Cancer J. 2015;21 4:254-62. doi:10.1097/ppo.0000000000000131.
6. Mumenthaler SM, Foo J, Choi NC, Heise N, Leder K, Agus DB, et al. The Impact of Microenvironmental Heterogeneity on the Evolution of Drug Resistance in Cancer Cells. Cancer Inform. 2015;14 Suppl 4:19-31. doi:10.4137/cin.s19338.
7. Straussman R, Morikawa T, Shee K, Barzily-Rokni M, Qian ZR, Du J, et al. Tumour micro-environment elicits innate resistance to RAF inhibitors through HGF secretion. Nature. 2012;487 7408:500-4. doi:10.1038/nature11183.
8. Zarour LR, Anand S, Billingsley KG, Bisson WH, Cercek A, Clarke MF, et al. Colorectal Cancer Liver Metastasis: Evolving Paradigms and Future Directions. Cell Mol Gastroenterol Hepatol. 2017;3 2:163-73. doi:10.1016/j.jcmgh.2017.01.006.
9. Burke D and Allen-Mersh TG. Colorectal liver metastases. Postgrad Med J. 1996;72 850:464-9. doi:10.1136/pgmj.72.850.464.
10. Bocuk D, Wolff A, Krause P, Salinas G, Bleckmann A, Hackl C, et al. The adaptation of colorectal cancer cells when forming metastases in the liver: expression of associated genes and pathways in a mouse model. BMC Cancer. 2017;17 1:342. doi:10.1186/s12885-017-3342-1.
11. Catarinella M, Monestiroli A, Escobar G, Fiocchi A, Tran NL, Aiolfi R, et al. IFNalpha gene/cell therapy curbs colorectal cancer colonization of the liver by acting on the hepatic microenvironment. EMBO Mol Med. 2016;8 2:155-70. doi:10.15252/emmm.201505395.
12. Van den Eynden GG, Majeed AW, Illemann M, Vermeulen PB, Bird NC, Hoyer-Hansen G, et al. The multifaceted role of the microenvironment in liver metastasis: biology and clinical implications. Cancer Res. 2013;73 7:2031-43. doi:10.1158/0008-5472.can-12-3931.
13. Quail DF and Joyce JA. Microenvironmental regulation of tumor progression and metastasis. Nat Med. 2013;19 11:1423-37. doi:10.1038/nm.3394.
14. Brown JM and Wilson WR. Exploiting tumour hypoxia in cancer treatment. Nature reviews Cancer. 2004;4 6:437-47. doi:10.1038/nrc1367.
15. Pickup MW, Mouw JK and Weaver VM. The extracellular matrix modulates the hallmarks of cancer. EMBO Rep. 2014;15 12:1243-53. doi:10.15252/embr.201439246.
16. Butcher DT, Alliston T and Weaver VM. A tense situation: forcing tumour progression. Nature reviews Cancer. 2009;9 2:108-22. doi:10.1038/nrc2544.
17. Mook OR, van Marle J, Jonges R, Vreeling-Sindelarova H, Frederiks WM and Van Noorden CJ. Interactions between colon cancer cells and hepatocytes in rats in relation to metastasis. Journal of cellular and molecular medicine. 2008;12 5b:2052-61. doi:10.1111/j.1582-4934.2008.00242.x.

18. Kaminska K, Szczylik C, Bielecka ZF, Bartnik E, Porta C, Lian F, et al. The role of the cell-cell interactions in cancer progression. Journal of cellular and molecular medicine. 2015;19 2:283-96. doi:10.1111/jcmm.12408.
19. Metzcar J, Wang Y, Heiland R and Macklin P. A Review of Cell-Based Computational Modeling in Cancer Biology. JCO Clin Cancer Inform. 2019;3:1-13. doi:10.1200/cci.18.00069.
20. Macklin P, Frieboes HB, Sparks JL, Ghaffarizadeh A, Friedman SH, Juarez EF, et al. Progress Towards Computational 3-D Multicellular Systems Biology. Advances in experimental medicine and biology. 2016;936:225-46. doi:10.1007/978-3-319-42023-3_12.
21. Macklin P. Key challenges facing data-driven multicellular systems biology. Gigascience. 2019;8 10 doi:10.1093/gigascience/giz127.
22. Zock JM. Applications of high content screening in life science research. Comb Chem High Throughput Screen. 2009;12 9:870-76.
23. Garvey CM, Spiller E, Lindsay D, Chiang CT, Choi NC, Agus DB, et al. A high-content image-based method for quantitatively studying context-dependent cell population dynamics. Sci Rep. 2016;6:29752. doi:10.1038/srep29752.
24. Chiang CT, Demetriou AN, Ung N, Choudhury N, Ghaffarian K, Ruderman DL, et al. mTORC2 contributes to the metabolic reprogramming in EGFR tyrosine-kinase inhibitor resistant cells in non-small cell lung cancer. Cancer Lett. 2018;434:152-9. doi:10.1016/j.canlet.2018.07.025.
25. Sullivan R, Pare GC, Frederiksen LJ, Semenza GL and Graham CH. Hypoxia-induced resistance to anticancer drugs is associated with decreased senescence and requires hypoxia-inducible factor-1 activity. Mol Cancer Ther. 2008;7 7:1961-73. doi:10.1158/1535-7163.mct-08-0198.
26. Hamada K, Monnai M, Kawai K, Nishime C, Kito C, Miyazaki N, et al. Liver metastasis models of colon cancer for evaluation of drug efficacy using NOD/Shi-scid IL2Rgammanull (NOG) mice. Int J Oncol. 2008;32 1:153-9.
27. Juarez EF, Lau R, Friedman SH, Ghaffarizadeh A, Jonckheere E, Agus DB, et al. Quantifying differences in cell line population dynamics using CellPD. BMC Syst Biol. 2016;10 1:92. doi:10.1186/s12918-016-0337-5.
28. Nagelkerke A, Bussink J, Rowan AE and Span PN. The mechanical microenvironment in cancer: How physics affects tumours. Semin Cancer Biol. 2015;35:62-70. doi:10.1016/j.semcancer.2015.09.001.
29. Baptista PM, Siddiqui MM, Lozier G, Rodriguez SR, Atala A and Soker S. The use of whole organ decellularization for the generation of a vascularized liver organoid. Hepatology. 2011;53 2:604-17. doi:10.1002/hep.24067.
30. Psaila B and Lyden D. The metastatic niche: adapting the foreign soil. Nature reviews Cancer. 2009;9 4:285-93. doi:10.1038/nrc2621.
31. Wei TT, Lin YT, Tang SP, Luo CK, Tsai CT, Shun CT, et al. Metabolic targeting of HIF-1alpha potentiates the therapeutic efficacy of oxaliplatin in colorectal cancer. Oncogene. 2019; doi:10.1038/s41388-019-0999-8.
32. Roberts DL, Williams KJ, Cowen RL, Barathova M, Eustace AJ, Brittain-Dissont S, et al. Contribution of HIF-1 and drug penetrance to oxaliplatin resistance in hypoxic colorectal cancer cells. Br J Cancer. 2009;101 8:1290-7. doi:10.1038/sj.bjc.6605311.
33. Ghaffarizadeh A, Heiland R, Friedman SH, Mumenthaler SM and Macklin P. PhysiCell: An open source physics-based cell simulator for 3-D multicellular systems. PLoS Comput Biol. 2018;14 2:e1005991. doi:10.1371/journal.pcbi.1005991.
34. Anderson AR, Rejniak KA, Gerlee P and Quaranta V. Microenvironment driven invasion: a multiscale multimodel investigation. J Math Biol. 2009;58 4-5:579-624. doi:10.1007/s00285-008-0210-2.

35. Gatenby RA, Smallbone K, Maini PK, Rose F, Averill J, Nagle RB, et al. Cellular adaptations to hypoxia and acidosis during somatic evolution of breast cancer. Br J Cancer. 2007;97 5:646-53. doi:10.1038/sj.bjc.6603922.
36. Drasdo D and Hohme S. A single-cell-based model of tumor growth in vitro: monolayers and spheroids. Phys Biol. 2005;2 3:133-47. doi:10.1088/1478-3975/2/3/001.
37. Cai Y, Xu S, Wu J and Long Q. Coupled modelling of tumour angiogenesis, tumour growth and blood perfusion. J Theor Biol. 2011;279 1:90-101. doi:10.1016/j.jtbi.2011.02.017.
38. Bauer AL, Jackson TL and Jiang Y. A cell-based model exhibiting branching and anastomosis during tumor-induced angiogenesis. Biophys J. 2007;92 9:3105-21. doi:10.1529/biophysj.106.101501.

Figure

Fig. 1. The impact of oxygen on CRC growth and treatment response to oxaliplatin.

Click here to download Figure Figures_Giga Science_v9.ppt

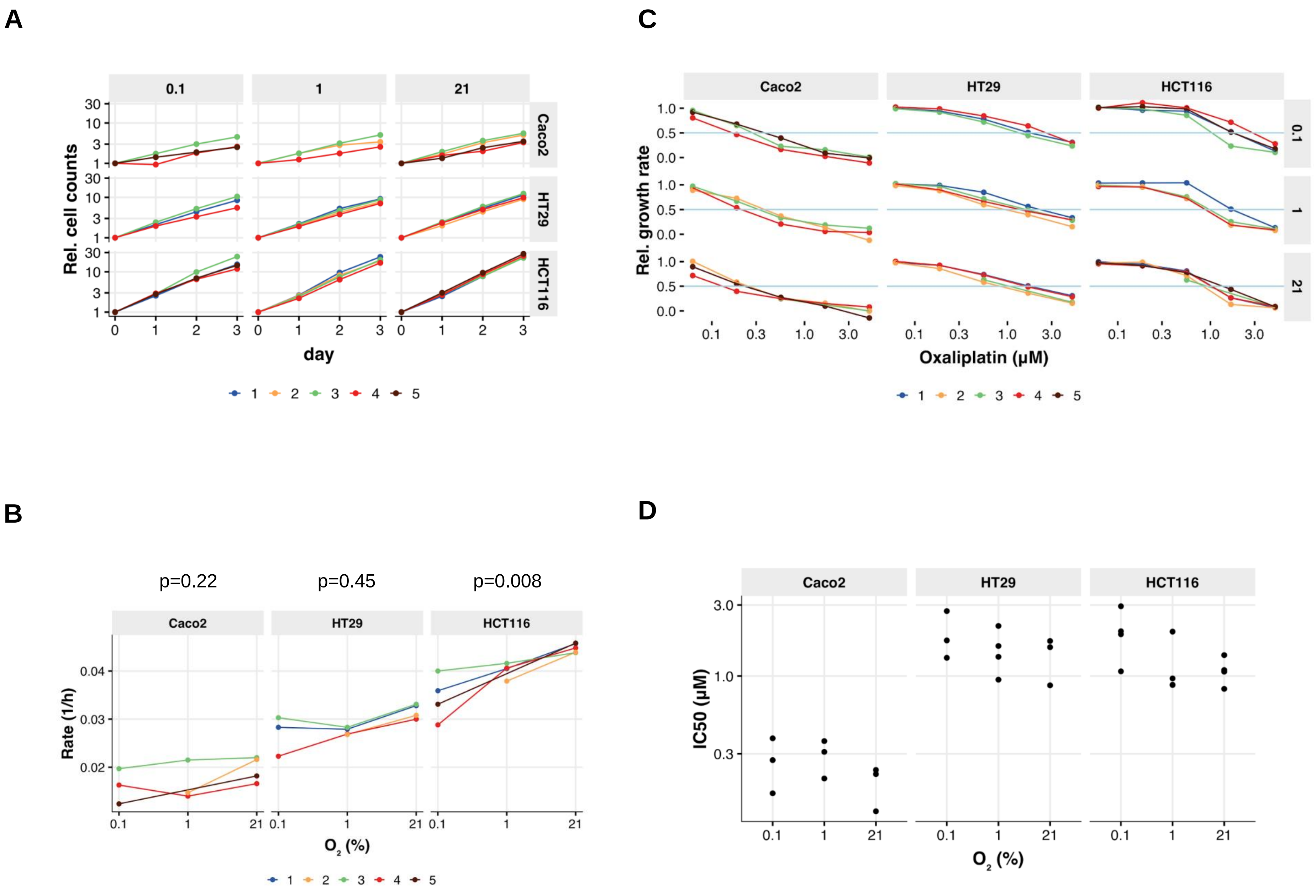

Fig. 2. The influence of stiffness on CRC growth and treatment response to oxaliplatin.

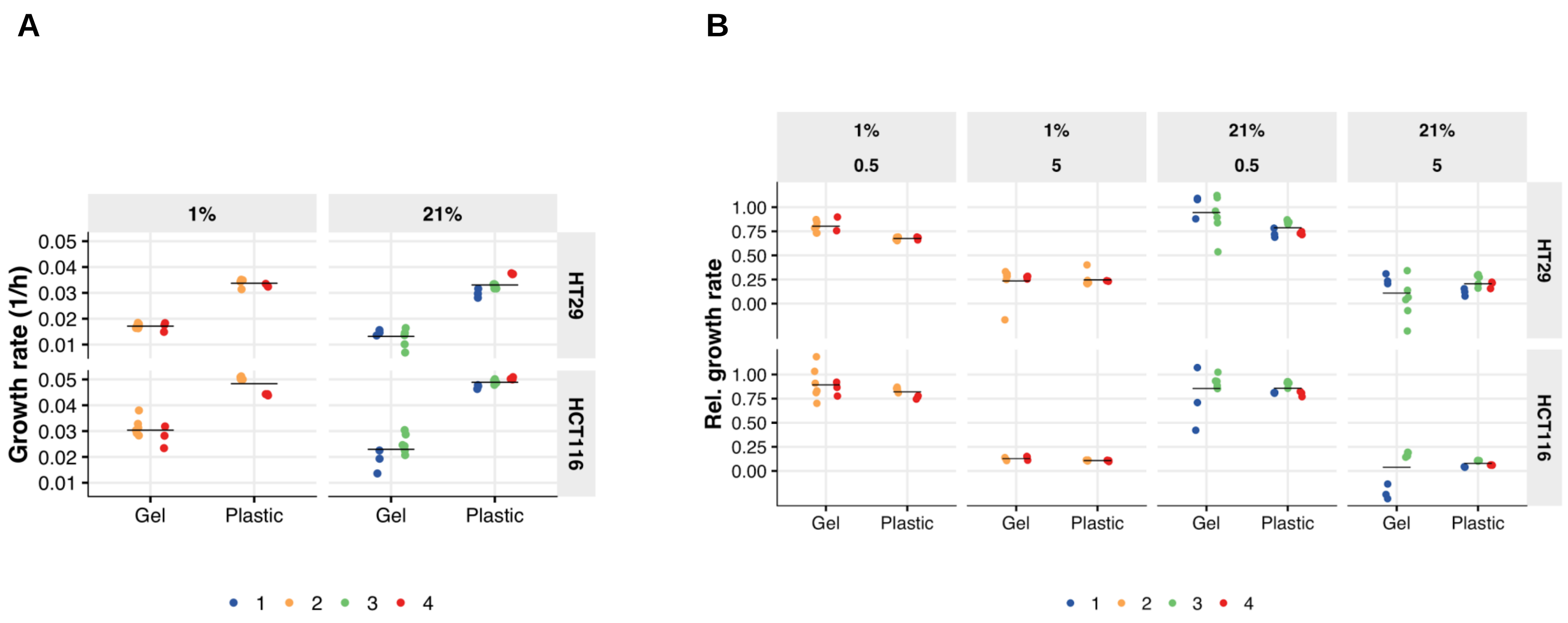

Fig. 3. The effect of liver ECM on the growth of CRC cells and treatment response to oxaliplatin.

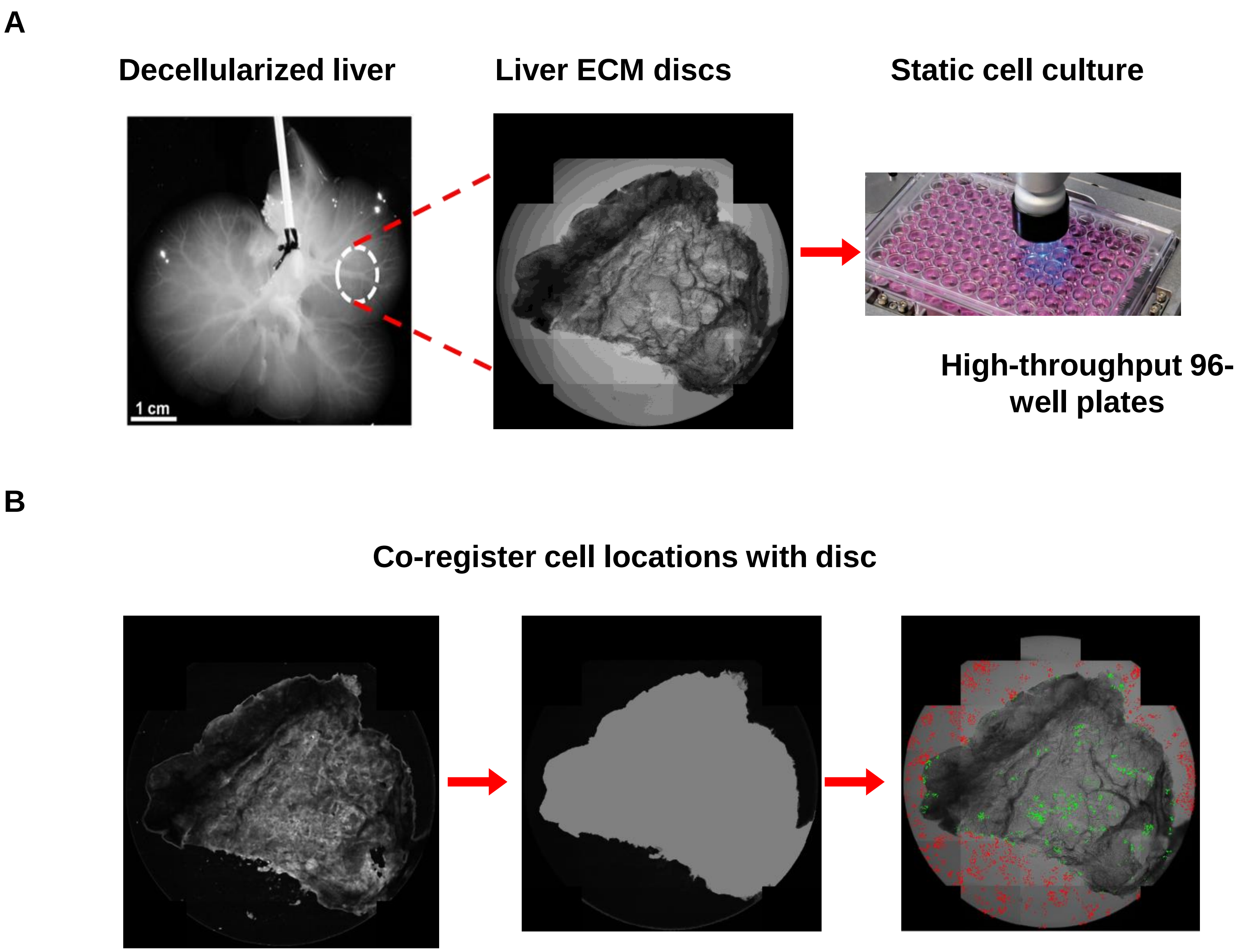

Fig. 3. The effect of liver ECM on the growth of CRC cells and treatment response to oxaliplatin.

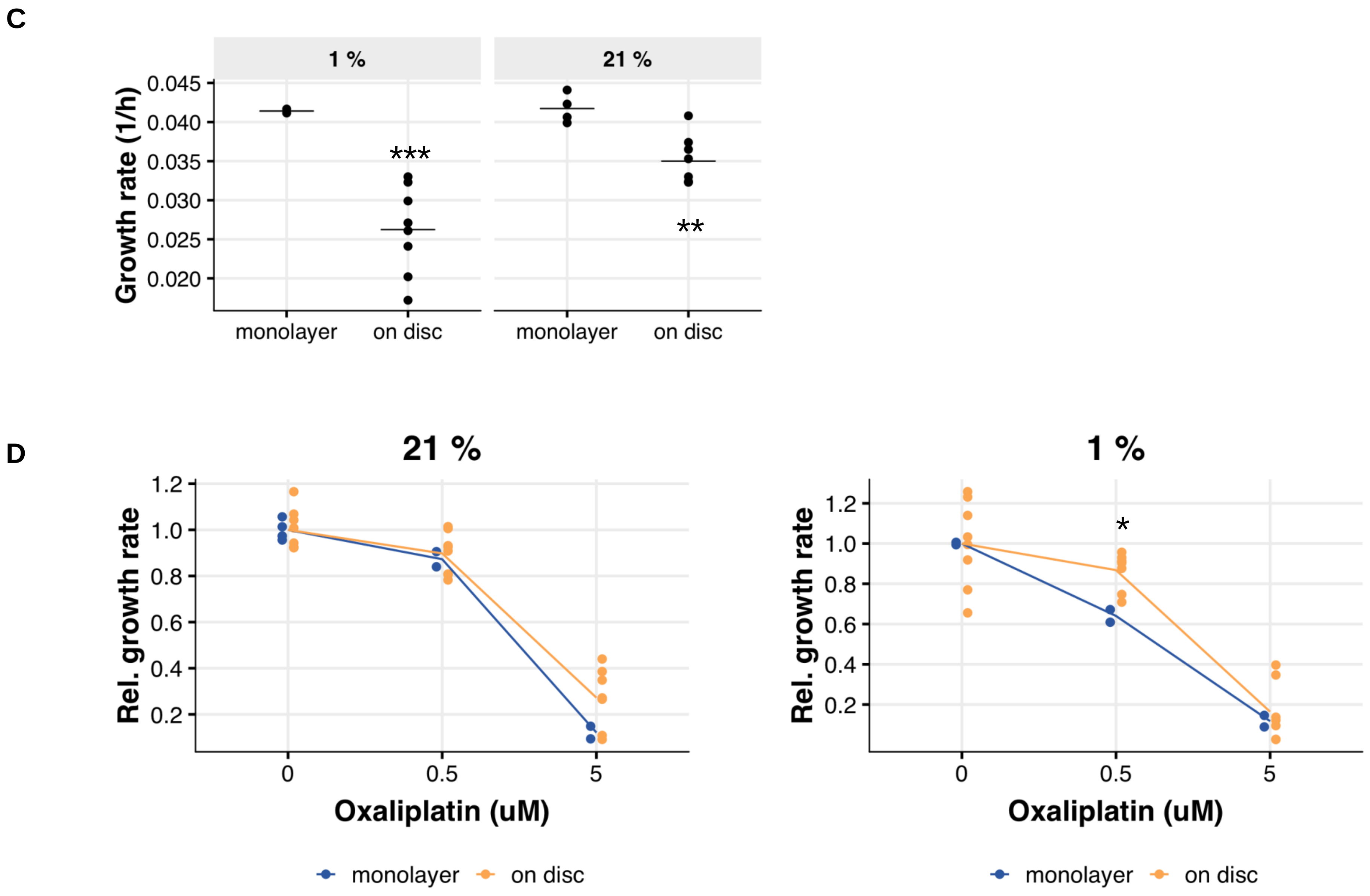

Click here to access/download
Supplementary Material
SupplementaryFigures_Gigascience_v9.ppt

Personal Cover
Click here to download Personal Cover CoverLetter_Gigascience_v4.pdf

Lawrence J. Ellison Institute for Transformative Medicine of USC

USC CENTER FOR APPLIED MOLECULAR MEDICINE
Shannon Mumenthaler, Ph.D.
Assistant Professor of Medicine

April 3rd, 2020

Dear Editor:

Please find enclosed our manuscript entitled "High throughput microscopy reveals the impact of multifactorial environmental perturbations on colorectal cancer cell growth" that we submit for consideration as a research article to the thematic series **Data-Driven Multicellular Systems Biology** in *Gigascience*.

Metastasis is the principle cause of cancer-related deaths. Developing novel treatments for metastatic diseases requires a systematic understanding of the multiparametric factors influencing tumor progression. Factors residing in the tumor microenvironment (TME) are considered to play a pivotal role in metastatic growth. However, traditional biological assays often only examine the impact of a single environmental factor in a qualitative manner. Leveraging the high-content imaging workflow we recently developed, we longitudinally measured the growth kinetics of colorectal cancer (CRC) cell lines influenced by various biochemical and biophysical microenvironmental contexts, specifically the combination of drug, oxygen, stiffness, and acellular liver extracellular matrix (ECM). We found highly aggressive HCT116 cells are affected by oxygen, substrate stiffness, and liver ECM. In addition, we showed that hypoxia has a protective effect against drug-induced cytotoxicity.

This expansive and quantitative data set of CRC cell growth measurements under *in situ* relevant environmental perturbations establishes a benchmark for data-driven computational modeling of cancer and other multicellular systems. These data provide insights into critical TME features contributing to metastatic seeding and tumor growth. They also provide a foundation for the development of computational models that can probe the relationships between growth dynamics, heterogeneous microenvironments, and the underlying biophysics of cancer metastasis. We believe our findings would fit the aims and scope of the thematic series **Data-Driven Multicellular Systems Biology**.

Thank you in advance for considering our work. All supporting data including raw images and scripts are transferring to the FTP server (ftp://user55@parrot.genomics.cn). All authors have read and approved the submission of this manuscript. This work is not under consideration for publication elsewhere and we have no conflicts of interest to disclose.

If you have any further questions, please don't hesitate to contact me.

Sincerely,

Shannon Mumenthaler

Shannon Mumenthaler, Ph.D.
Assistant Professor of Medicine

University of Southern California
2250 Alcazar Street, CSC 240, Mail Code 9075, Los Angeles, California 90033 • Tel: 323 442 2529 • Fax: 323 442 2764 • smumenth@usc.edu
