# Supplementary material for: High-throughput microscopy reveals the impact of multifactorial environmental perturbations on colorectal cancer cell growth
Source: Gigascience. 2021 Apr 19;10(4):giab026. doi: 10.1093/gigascience/giab026 (PMC8054261; doi:10.1093/gigascience/giab026)
Supplement: giab026_GIGA-D-20-00103_Revision_1 [file giab026_giga-d-20-00103_revision_1.pdf]

GigaScience

High throughput microscopy reveals the impact of multifactorial environmental perturbations on colorectal cancer cell growth

--Manuscript Draft--

**Manuscript Number:**	GIGA-D-20-00103R1
**Full Title:**	High throughput microscopy reveals the impact of multifactorial environmental perturbations on colorectal cancer cell growth
**Article Type:**	Research
**Funding Information:**	National Cancer Institute (R01CA180149) — Dr. David B. Agus
**Abstract:**	Abstract<br>Background: Colorectal cancer (CRC) mortality is principally due to metastatic disease, with the most frequent organ of metastasis being liver. Biochemical and mechanical factors residing in the tumor microenvironment (TME) are considered to play a pivotal role in metastatic growth and response to therapy. However, it is difficult to study the TME systematically due to a lack of fully controlled model systems that can be investigated in rigorous detail.<br>Results: We present a quantitative imaging dataset of CRC cell growth dynamics influenced by in vivo-mimicking conditions. They consist of tumor cells grown in various biochemical and biomechanical microenvironmental contexts. These contexts include varying oxygen and drug concentrations, and growth on conventional stiff plastic, softer matrices, and bioengineered acellular liver extracellular matrix (ECM). Growth rate analyses under these conditions were performed via the cell phenotype digitizer (CellPD).<br>Conclusions: Our data indicate the growth of highly aggressive HCT116 cells is affected by oxygen, substrate stiffness, and liver ECM. In addition, hypoxia has a protective effect against oxaliplatin-induced cytotoxicity on plastic and liver ECM. This expansive data set of CRC cell growth measurements under in situ relevant environmental perturbations provides insights into critical TME features contributing to metastatic seeding and tumor growth. Such insights are essential to dynamical modeling and understanding the multicellular tumor-stroma dynamics that contribute to metastatic colonization. It also establishes a benchmark data set for training and testing data-driven dynamical models of cancer cell lines and therapeutic response in a variety of microenvironmental conditions.
**Corresponding Author:**	Shannon Mumenthaler<br>University of Southern California Health Sciences Center<br>Los Angeles, California UNITED STATES
**Corresponding Author Secondary Information:**	
**Corresponding Author's Institution:**	University of Southern California Health Sciences Center
**Corresponding Author's Secondary Institution:**	
**First Author:**	Chun-Te Chiang, Ph.D.
**First Author Secondary Information:**	
**Order of Authors:**	Chun-Te Chiang, Ph.D.
	Roy Lau
	Ahmadreza Ghaffarizadeh, Ph.D.
	Matthew Brovold
	Dipen Vyas, Ph.D.
	Edwin F Juárez, Ph.D.
	Anthony Atala, M.D.

Powered by Editorial Manager® and ProduXion Manager® from Aries Systems Corporation

	David B. Agus, M.D.
	Shay Soker, Ph.D.
	Paul Macklin, Ph.D.
	Daniel Ruderman, Ph.D.
	Shannon M Mumenthaler, PhD
**Order of Authors Secondary Information:**	
**Response to Reviewers:**	Summary of Response to Reviewers<br><br>General Comments: We greatly appreciate the comments of the reviewers and find that their suggestions have made this a much stronger manuscript. We have addressed each point in a revised manuscript and have documented the major edits under “Actions taken.”<br><br>Response to reviewer #1<br><br>This excellent Research Article utilises a quantitative imaging approach to elucidate the effects of hypoxia, chemotherapeutics, and extracellular matrix stiffness in an in vitro model of colorectal cancer. Three different colorectal cancer cell lines were used in this study and growth rates were quantified using an Operetta High Content Imaging system. I was particularly intrigued by the novel use of decellularized liver scaffolds as a means of assessing the role of tissue architecture on colorectal tumor cell growth. The image analysis reported in this study highlights that growth kinetics of colorectal tumour cells on decellularized liver scaffolds is "markedly different" from plastic cell culture plates, and that HCT116-H2BGFP cells cultured on liver ECM were less sensitive to oxaliplatin treatment under hypoxic conditions. From a biomedical perspective, where the tropism of metastatic colorectal cancer cells for liver is well established, I consider this finding especially relevant.<br><br>The supporting image data for this study have been submitted to GigaScience. These data are extremely well organised and I thank the authors for providing details of: row, column, field, plane, channel, and time point for each of the 16-bit images used in this study. In addition, the supporting analyses have also been submitted to GigaScience as tabular data, as have the supporting scripts.<br><br>I accept this article for publication in GigaScience. Furthermore, there is a clear need for benchmark datasets of this nature, and I fully support the authors' request to include this manuscript in the GigaScience thematic series entitled "Data-Driven Multicellular Systems Biology."<br><br>We thank the reviewer for their valuable assessment of our manuscript and appreciate the feedback that was provided.<br><br>Response to reviewer #2<br><br>The article is simple, straightforward and easy to follow. The ability of high content screening to measure phenotypic changes with alterations to the tumor microenvironment such as hypoxia and matrix stiffness has been studied using 3 different cell lines. In addition to modifying oxygen levels, the authors also perform co-cultures with an interesting acellular liver ECM disk to observe the effect of liver scaffolds in tumor growth patterns.<br><br>We thank Reviewer 2 for the positive assessment of our manuscript and the constructive comments for improvement. Due to the coronavirus and university shut-downs, we were unable to perform additional liver ECM disc and animal studies. However, we have carefully addressed the reviewer’s additional comments with new softwell experiments and modifications to the text in this revised version of the manuscript and in a point-by-point response below.<br><br>While figures 1-2 report changes in multiple cell lines, figure 4 highlights the effect of

liver scaffolds on only the most aggressive and metastatic cell line HCT116. Reporting the effect of liver scaffold on the growth of less aggressive and metastatic cell lines and comparing and contrasting the differences would have been more effective at studying ECM based regulation of tumor growth. The authors could also have utilized more fluorescent markers in addition to DAPI to determine the effect of the hypoxia and liver ECM on tumor biology.

We thank the reviewer for pointing this out. Given HCT116 is one of the most efficient CRC cell lines to metastasize to liver in different animal models, we prioritized the measurements of this line in response to the microenvironmental perturbation on the liver ECM discs. We agree with the reviewer that investigating why certain cell lines do not grow well in the liver is a very interesting and important topic to study. While a detailed assessment is beyond the scope of this manuscript; we did perform additional softwell experiments that more closely mimic the tissue stiffness of liver metastases (LM) (2 kPa) to quantify the growth capabilities of HCT116 and HT29 cells. We found HCT116 cells grow faster on the 2 kPa stiffness that is more similar to LM than on the 0.2 kPa stiffness which is more similar to the primary colon tumor, but this was not observed in the less aggressive HT29 cell line. We believe these data provide important insights for future studies to compare the differential adaptation of CRC cells to the liver microenvironment. Interestingly, we also found the LM stiffness has a protective effect against low dose oxaliplatin-induced cytotoxicity of HT29 cells under 1% oxygen concentration; highlighting the multiplexing capabilities of high content screening. We further discuss the potential for incorporation of additional markers for cell phenotyping using high content screening.

Actions taken
Figs. 2A and 2B > Added 3 biological replicate experiments and revised figures with a side-by-side comparison of the growth of HCT116 and HT29 cells and treatment response to oxaliplatin on 0.2 kPa softwell (mimics tissue stiffness of primary colon tumor), 2 kPa softwell (mimics tissue stiffness of LM) and conventional plastic (3 GPa). All additional raw data files have been uploaded to GigaDB.

Results > Added citation Shen et al. (ref25) and mentioned that 0.2 kPa and 2 kPa are close to the stiffness of primary CRC and liver metastases, respectively. Summarized the results of HCT116 and HT29 cells in response to different stiffness.

Results > Added citations Xu et al. (ref40) and Ishizu et al. (ref41). Revised text to point out that HCT116 is the most efficient line to develop liver metastasis in orthotopic and ectopic animal models to support the choice for examining HCT116 in the liver ECM experiments.

Discussion > Added citation Ishizu et al. (ref41). Discussed the stiffer LM microenvironment may also contribute to the aggressive phenotypes of HCT116 cells.

Discussion > Added citations You et al. (ref46) and Wouters et al. (ref47). Discussed the potential use of other markers to further interrogate the relationship between hypoxia and stiffness in drug sensitivity.

Did the authors test any more chemotherapeutic drugs other than oxaliplatin ? And, what about testing combinations of drugs under these culture conditions ?
Were there plans to implant the cell lines grown in co-cultures or normoxic conditions in mice to follow tumor growth ?

We thank the reviewer for the comment and suggestion. We chose oxaliplatin because it is one of the most effective cytotoxic compounds used in the adjuvant and advanced setting of CRC treatment. We agree with the reviewer that testing additional drugs and drug combinations both in vitro and in vivo would be interesting; however, we believe these studies are beyond the scope of this manuscript and best suited to include in a follow-up manuscript.

Actions taken
Results > Added citation Alcindor et al. (ref37). Revised text to emphasize that oxaliplatin is commonly used in the clinical setting.

Fig 3B (what's the purpose of the yellow arrow mark?)

Thank you for making this point. We are sorry that our presentation may have been confusing. The purpose of the arrow was to connect steps (1), (2), (3) to demonstrate how we segment the cells on the discs.

Actions taken
Figures> Labeled steps 1, 2, and 3 in Fig. 3B to avoid confusion.

Response to reviewer #3

The manuscript High throughput microscopy reveals the impact of multifactorial environmental perturbations on colorectal cancer cell growth and shows that HCT116 cell growth is affected by oxygen, substrate stiffness, and liver ECM. This data set could be set for training and testing data driven computational models of cancer and other multicellular systems.
The manuscript is well written however, there are some major issues I have with the manuscript:

We appreciate Reviewer 3’s thorough review of our manuscript and valuable comments. We are delighted that the reviewer found our findings very interesting. The reviewer feedback has been instrumental in improving the overall impact of our manuscript. We have addressed each concern in a revised manuscript and have documented the changes in a point-by-point response below.

1. There is no stiffness mention in the background. It is only mentioned briefly in one sentence, but it is essential for your results and it is in the conclusion of the abstract. There should be at least a paragraph about stiffness in the background.

We thank the reviewer for pointing this out and have added a paragraph in the introduction to emphasize the importance of tissue stiffness in the tumor microenvironment and to address the relationship between stiffness and liver metastasis in colorectal cancer.

Actions taken
Introduction > Added citations Cox et al. (ref19), Mohammadi et al. (ref20), Nagelkerke et al. (ref22), Laklai et al. (ref23), Colpaert et al. (ref24), and Shen et al. (ref25). Included text regarding stiffness and its relationship with liver metastasis.

2. Why using 0.2kPa and 3GPa in the experiments? Those two values are not physiological stiffness. It would be useful to do those experiments with physiological values for example the kPa of a tumor and a normal cell and the 3GPa.

We appreciate the reviewer bringing up the physiological stiffness values in cancer. Based on a recent Cancer Cell paper (PMID: 32516590), the stiffness of liver metastases in metastatic colorectal cancer is significantly higher compared with the primary tumor. The stiffness of primary colorectal cancer is close to 0.2 kPa, and the stiffness of liver metastases is around 2 kPa. Therefore, we performed additional experiments to measure the growth and treatment response of colorectal cancer cells in a 2 kPa environment.

Actions taken
Figs. 2A and 2B > Added 3 biological replicate experiments and revised figures with a side-by-side comparison of the growth of HCT116 and HT29 cells and treatment response to oxaliplatin on 0.2 kPa softwell (mimics tissue stiffness of primary colon tumor), 2 kPa softwell (mimics tissue stiffness of LM) and conventional plastic (3 GPa). All additional raw data files have been uploaded to GigaDB.

Results > Added citation Shen et al. (ref25) and mentioned that the stiffness of 0.2 kPa and 2 kPa are close to the stiffness of primary CRC and liver metastasis, respectively. Summarized the results of HCT116 and HT29 cells in response to different stiffness.

3. Discussion needs further editing.

Thank you for making this point. We added discussion points regarding the differential response to hypoxia between HCT116 and HT29 cells and whether this might be due to differences in p53 and MSI status. In addition, we discussed the differential adaptation of CRC cells to the liver microenvironment. We also discussed potential pathway analyses that can be further explored in future mechanistic and modeling studies. We also carefully reviewed to correct for any previous grammatical errors.

Actions taken
Discussion > Added citations Toscano et al. (ref43), Berg et al. (ref44), Ahmed et al. (ref45), and Scanlon et al. (ref16). Discussed the differential response to hypoxia between HCT116 and HT29 cells.

Discussion > Added citations Ishizu et al. (ref41). Discussed the stiffer LM microenvironment may also contribute to the aggressive phenotypes of HCT116 cells.

Discussion > Added citations You et al. (ref46) and Wouters et al. (ref47). Discussed the relationship between hypoxia and stiffness in drug sensitivity.

Discussion > Added citation Wang et al. (ref54). Discussed how the phenotypic parameters generated from this study could motivate new biological hypotheses.

4. HT-29 are MSS and HCT116 are MSI. There is some literature about hypoxia and MSI which could be discussed and mentioned in the background

This is an excellent suggestion. We have mentioned the relationship between hypoxia and MSI in the introduction and discussed the potential impact of MSI status on the differential results observed between HCT116 and HT29.

Actions taken
Introduction > Added citations Gelman et al. (ref14), Brown et al. (ref15), Scanlon et al. (ref16), and Boland et al. (ref17) to show hypoxia is particularly pervasive in the liver microenvironment and hypoxia could reduce DNA mismatch repair gene expression and enrich for mismatch repair deficient cells.

Discussion > Added citations Toscano et al. (ref43), Berg et al. (ref44), and Ahmed et al. (ref45), and Scanlon et al. (ref16) to highlight that HCT116 and HT29 cells are MSI and MSS, respectively and to discuss the differential response to hypoxia between HCT116 and HT29 cells.

5. Figure 1 to figure 2 needs a better transfer why the author is doing this experiment.

We thank the reviewer for this comment and agree that there is a connection between hypoxia and stiffness that should be mentioned as a transition from Fig.1 to Fig. 2.

Actions taken
Results > Added citations Laklai et al. (ref23) and Erler et al. (ref38) and added text describing how hypoxia can modulate tumor progression through increased ECM crosslinking and stiffening. Also added citation Shen et al. (ref25) to demonstrate that increased stiffness in liver metastases plays a pivotal role in CRC progression.

6. To really say that this data set could be used for training and testing data driven computational models of cancer and other multicellular systems it would need more than 3 cell lines to perform these experiments. Also, some human data would be useful to make this statement.

Thank you to the reviewer for making this point. The purpose of this study is not to train an AI model across many cell types, but rather use this type of data to calibrate mechanistic, dynamical simulation models of individual cell types. Also, the cell lines used in this manuscript (HCT116, HT29, and Caco2) are often used in such multidisciplinary studies. We modified the abstract to avoid confusion and further edited the discussion on these points. We note that the experiments presented in this manuscript provided sufficient data to determine the net cell proliferation rate for each cell line under a broad variety of oxygenation, ECM, and oxaliplatin treatment

	conditions. These can be directly incorporated into dynamical models, as is planned for follow-up studies. Moreover, each cell line's plotted curves of proliferation versus O2 (Fig. 1b), proliferation versus drug (Fig. 1c), IC50 versus O2 (Fig. 1D), growth versus ECM stiffness and drug (Fig. 2), and cell proliferation on ECM discs (Fig. 3) will be of great benefit to mathematical biologists as they postulate biological hypotheses to drive refined dynamical models.<br><br>Actions taken<br>Abstract > Edited "data-driven computational models of cancer and other multicellular systems" to "data-driven dynamical models of cancer cell lines and therapeutic response in a variety of microenvironmental conditions."<br><br>Discussion > Added citation Wang et al. (ref54). Discussed how the phenotypic parameters generated from this study could motivate new biological hypotheses.
**Additional Information:**	
**Question**	**Response**
Are you submitting this manuscript to a special series or article collection?	No
**Experimental design and statistics**<br><br>Full details of the experimental design and statistical methods used should be given in the Methods section, as detailed in our Minimum Standards Reporting Checklist. Information essential to interpreting the data presented should be made available in the figure legends.<br><br>Have you included all the information requested in your manuscript?	Yes
**Resources**<br><br>A description of all resources used, including antibodies, cell lines, animals and software tools, with enough information to allow them to be uniquely identified, should be included in the Methods section. Authors are strongly encouraged to cite Research Resource Identifiers (RRIDs) for antibodies, model organisms and tools, where possible.<br><br>Have you included the information requested as detailed in our Minimum Standards Reporting Checklist?	Yes
**Availability of data and materials**	Yes

All datasets and code on which the conclusions of the paper rely must be either included in your submission or deposited in publicly available repositories (where available and ethically appropriate), referencing such data using a unique identifier in the references and in the "Availability of Data and Materials" section of your manuscript.<br><br>Have you have met the above requirement as detailed in our Minimum Standards Reporting Checklist?	

Powered by Editorial Manager® and ProduXion Manager® from Aries Systems Corporation

Manuscript
Click here to access/download;Manuscript;Manuscript_Gigascience_revision_

High throughput microscopy reveals the impact of multifactorial environmental perturbations on colorectal cancer cell growth

Chun-Te Chiang[1*], Roy Lau[1*], Ahmadreza Ghaffarizadeh[1], Matthew Brovold[2], Dipen Vyas[2], Edwin F. Juárez[1], Anthony Atala[2], David B. Agus[1], Shay Soker[2], Paul Macklin[1,3], Daniel Ruderman[1ł], Shannon M. Mumenthaler[1ł]

[1] Lawrence J. Ellison Institute for Transformative Medicine, University of Southern California, Los Angeles, California, USA
[2] Wake Forest Institute for Regenerative Medicine, Winston Salem, North Carolina, USA
[3] Intelligent Systems Engineering, Indiana University, Bloomington, IN, USA
[*] These authors contributed equally to this work
[ł] Co-corresponding authors

Corresponding Authors

D.R.: Department of Medicine, University of Southern California, 2250 Alcazar Street, CSC 240, Los Angeles, CA 90033. Phone: (323) 442-2839. E-mail: ruderman@usc.edu

S.M.: Department of Medicine, University of Southern California, 2250 Alcazar Street, CSC 242, Los Angeles, CA 90033. Phone: (323) 442-2529. E-mail: smumenth@usc.edu

ORCIDs:

Chun-Te Chiang, 0000-0003-0345-2400
Roy Lau, 0000-0001-8405-1689
Ahmadreza Ghaffarizadeh, 0000-0002-9359-8800
Edwin F. Juárez, 0000-0003-1062-3642
Anthony Atala, 0000-0001-8186-2160
David B. Agus, 0000-0002-7499-7822
Shay Soker, 0000-0002-8458-9232
Paul Macklin, 0000-0002-9925-0151
Daniel Ruderman, 0000-0001-5787-5009
Shannon M Mumenthaler, 0000-0003-2687-3769

Abstract

Background: Colorectal cancer (CRC) mortality is principally due to metastatic disease, with the most frequent organ of metastasis being liver. Biochemical and mechanical factors residing in the tumor microenvironment (TME) are considered to play a pivotal role in metastatic growth and response to therapy. However, it is difficult to study the TME systematically due to a lack of fully controlled model systems that can be investigated in rigorous detail.

Results: We present a quantitative imaging dataset of CRC cell growth dynamics influenced by *in vivo*-mimicking conditions. They consist of tumor cells grown in various biochemical and biomechanical microenvironmental contexts. These contexts include varying oxygen and drug concentrations, and growth on conventional stiff plastic, softer matrices, and bioengineered acellular liver extracellular matrix (ECM). Growth rate analyses under these conditions were performed via the cell phenotype digitizer (CellPD).

Conclusions: Our data indicate the growth of highly aggressive HCT116 cells is affected by oxygen, substrate stiffness, and liver ECM. In addition, hypoxia has a protective effect against oxaliplatin-induced cytotoxicity on plastic and liver ECM. This expansive data set of CRC cell growth measurements under *in situ* relevant environmental perturbations provides insights into critical TME features contributing to metastatic seeding and tumor growth. Such insights are essential to dynamical modeling and understanding the multicellular tumor-stroma dynamics that contribute to metastatic colonization. It also establishes a benchmark data set for training and testing data-driven dynamical models of cancer cell lines and therapeutic response in a variety of microenvironmental conditions.

Keywords: Colorectal Cancer, High Content Imaging, Liver Metastasis, Tumor Microenvironment

Background

Colorectal cancer (CRC) is the third most deadly cancer in both men and women in the United States [1]. Current treatment strategies include FOLFOX (5-FU, leucovorin and oxaliplatin), FOLFIRI (5-FU, leucovorin and irinotecan) or XELOX (oxaliplatin and capecitabine) with or without molecular targeted drugs [2]. The 5-year survival rate for CRC is 90% if the cancer is diagnosed locally. However, once the disease has spread to distant sites, this rate drops dramatically to around 10% [3]. Emerging data, spanning from clinical to laboratory research, highlights that metastatic disease cannot be explained solely by the genetics of the cancer cells; instead,

bidirectional interactions with the surrounding microenvironment play a pivotal role in tumor progression [4-7]. Devising innovative ways to treat CRC metastasis needs to address not only the genetic heterogeneity of the tumor but also its dynamic microenvironment.

Liver is the most common organ of distant metastasis. More than 50% of CRC patients with advanced disease develop liver metastases [8]. Many such metastases are discovered months or years after initial seeding. Consequently, most patients require treatment for established liver tumor foci. It has been postulated that the high rate of liver metastases is a result of anatomical considerations, with the portal vein draining directly from the colon and upper rectum to the liver [9]. However, increasing evidence has demonstrated that the liver microenvironment is vital in influencing CRC metastasis [10-12]. Low oxygen levels (hypoxia) are a key component in the liver tumor microenvironment (TME) [13]. The oxygen concentration in the portal vein is around 1% [14]. Poor vascularization in the tumor mass can further promote an oxygen-limited environment and initiate more aggressive phenotypes [15]. Hypoxia can lead to genomic instability by influencing DNA repair pathways [16]. Specifically, hypoxia has been shown to down-regulate the expression of DNA mismatch repair proteins at both transcriptional and translational levels [16]. Alterations in the mismatch repair machinery are known to result in microsatellite instability (MSI), which is found in approximately 15% of CRC [17].

Altered extracellular matrix (ECM) has also been considered a key feature of the TME [18]. ECM physically supports tissues and provides a substrate for cell adhesion and migration [19]. Cellular interactions with the surrounding ECM can regulate a vast range of biological outcomes including disease progression and drug resistance [20]. Different tissues are known to have distinct ECM molecular compositions and architectures. The complexity of the ECM is not merely biochemical: associated mechanical properties, like stiffness, can also greatly impact cell proliferation and motility [21]. It is well known that tumor tissue is much stiffer than surrounding normal tissues [22]. Increased tissue stiffness can influence tumor growth, metabolism, invasion, and metastasis and has been demonstrated to play a significant role in disease progression of several solid tumors and to correlate with patient outcomes [23, 24]. A recent study showed that the stiffness of liver metastases is significantly higher than that of primary CRC tumors and that the metastatic stiffness is closely correlated with tissue vascularity [25]. Moreover, adhesive tumor cell interactions with liver cells—particularly endothelial cells and hepatocytes in the sinusoids—have recently been shown to impact metastatic progression [26] as well as chemical communication with stellate, Kupffer, and inflammatory cells [27], illustrating the importance of ECM stiffness as a parameter to consider in studying CRC progression.

The TME is a highly complex system with many factors working in concert. However, traditional biological assays often only examine a single environmental factor in a qualitative way, and thereby lack the ability to recapitulate essential features of metastatic growth. Multicellular computational modeling can provide novel insights to link cancer progression to heterogeneous TME conditions and the dynamical interactions between tumor cells and the resident cell ecosystem [28, 29]. However, predicting the impact of liver microenvironmental manipulations on CRC behavior requires high-quality benchmark datasets to fit model parameters and drive the development of computational models [30], particularly systems that can independently and separately study the role of metastatic tumor cell interactions with hypoxia, the ECM, and resident liver cells. High content screening (HCS), the application of automated microscopy and image analysis, has been widely used in cell biology and drug development to screen drug compounds for safety and toxicity on human cells *in vitro* [31]. This platform is also well suited for exploring the impact of multiple TME parameters, either individually or simultaneously, on cell behavior [32, 33]. Here we extend the use of our previously established imaging workflow to study the

biophysical and biochemical impact of the organ context, specifically the combination of oxygen, stiffness, and liver ECM, on CRC cell growth and response to therapy. After isolating the impact of hypoxia and ECM biomechanics on CRC cell seeding, we can control for these factors and continue high-throughput investigations that isolate and characterize adhesive and other multicellular interactions during metastatic colonization.

Results

CRC growth rates under different oxygen tensions

Tumor hypoxia, or oxygen deprivation, has been shown to decrease proliferation and limit cells' responsiveness to therapeutic agents [34]. To investigate the impact of hypoxia on CRC growth, we examined three human CRC cell lines with different aggressiveness (Caco2; HT29; HCT116) grown under various oxygen concentrations (normoxic-21%; hypoxic-1% and 0.1%). HCT116 was derived from a poorly differentiated human colon adenocarcinoma known to develop hepatic metastases efficiently in immunodeficient mice. HT29 also has metastatic capabilities but less efficient compared with HCT116. In contrast, Caco2 shows no ability to metastasize [35]. We used the Operetta high content imaging system and Harmony software (Version 3.5.2) to measure cell counts of CRC cell lines grown in the respective oxygen conditions (Fig. 1A and S1). To extract growth rates from sequential cell count data, we utilized CellPD (cell phenotype digitizer) (Version 1.0.1), a previously developed open source Python code which leverages the Levenberg-Marquardt algorithm to perform nonlinear least squares minimization between simulated and experimental cell counts [36]. We observed a trend toward reduced growth rates under the hypoxic conditions in the most aggressive cell line HCT116 ($p=0.008$), which is not present in the less aggressive cell lines, HT29 and Caco2 (Fig. 1B).

The impact of hypoxia on drug response

Oxaliplatin is a standard CRC chemotherapy agent used in the adjuvant and advanced setting [37]. To examine the effect of oxygen on cancer treatment response, we measured CRC growth effects of oxaliplatin under different oxygen concentrations (various concentrations of oxygen – 20, 1, 0.1% and oxaliplatin treatment – 0, 0.062, 0.185, 0.555, 1.667, 5 μM). We found the least aggressive cell line, Caco2, to be most sensitive to oxaliplatin treatment compared to HT29 and HCT116 across all oxygen concentrations (Fig. 1C). Interestingly, the IC_{50} of oxaliplatin was not significantly altered under hypoxia in both Caco2 and HT29 cells. However, there is a difference in the IC_{50} of oxaliplatin between 0.1 and 21% oxygen with a mean estimate of 1.7-fold increase (95% credible interval (95%CI): 1.02 to 2.97) in HCT116 cells (Fig. 1D).

Matrix stiffness environment influences CRC cell growth

A hypoxic tumor environment can lead to the overexpression of ECM proteins by tumor cells, increasing the crosslinking and stiffening of the ECM [38]. The dense fibrotic matrix in solid tumors contributes to local hypoxia through elevating interstitial fluid pressure and disrupting neovascularization [23]. Recent evidence demonstrates that increased stiffness plays a pivotal role in CRC progression and metastasis [25]. Traditional approaches to measure stiffness effects on cancer cell growth involves culturing cells in Matrigel or soft agar; however, their mechanical properties are poorly defined. Here we cultured cells on commercially available collagen-coated polyacrylamide plates (Softwell) with stiffness of 0.2k Pa and 2 kPa (mimicking the stiffness of primary CRC tumors and liver metastases, respectively) [25]. To longitudinally measure cell growth on the softwells, we generated fluorescently labeled HCT116 and HT29 cells through stable infection with Histone-2B-GFP lentiviruses (HCT116-H2BGFP and HT29-H2BGFP) (Fig. S2A). To verify the transfection did not alter cell behavior, growth rates of HCT116-H2BGFP and HT29-H2BGFP were compared to unlabeled cells (Fig. S2B). For subsequent investigations into

TME-induced cell phenotypes, we used the HCT116-H2BGFP and HT29-H2BGFP cells. Growth rates were evaluated from cells cultured on 0.2 kPa softwell, 2 kPa softwell, and conventional plastic (~3GPa) plates using time series data consisting of live cell counts obtained over a period of 0-72 hours. We found softer matrices (0.2 kPa and 2 kPa softwell) reduced the growth rate of both cell lines under 1 and 21% oxygen concentrations (Fig. 2A). We observed an increased growth rate of HCT116-H2BGFP cells under 21% oxygen conditions on 2.0 kPa stiffness compared to 0.2 kPa with a posterior mean estimate of 0.006 (95% CI: 0.001 to 0.011) per hour (Fig. 2A). There was no measured difference (95% CI contains 0) in growth rate between 0.2 kPa and 2 kPa in HT29-H2BGFP cells (Fig. 2A). The sensitivity to oxaliplatin was not altered by stiffness in HCT116 cells, however, a protective effect by softer matrices was observed in HT29-H2BGFP cells under a low dose of oxaliplatin treatment in 21% and 1% oxygen environment with a mean estimate of 0.21 (95% CI: 0.08 to 0.33) and 0.16 (95% CI: 0.02 to 0.29), respectively (Fig.2B). In addition, compared to 0.2 kPa, we found 2.0 kPa increased the relative growth rate of low-dose oxaliplatin-treated HT29-H2BGFP cells under 1% oxygen concentrations with a posterior mean estimate of 0.23 (95% CI: 0.03 to 0.41) (Fig. 2B).

CRC cell growth on liver ECM

The ECM is an essential, yet understudied, component of the tumor microenvironment that physically supports tissues and provides a substrate for cell adhesion and migration, as well as a source of bioactive molecules [18]. To further interrogate the interaction of tumor cells with the metastatic tissue microenvironment, we developed a model of metastatic CRC growth in the liver using acellular liver ECM following our previously published detergent-based perfusion technique [39]. The decellularized liver scaffolds maintain important native ECM components such as collagens, laminin and fibronectin, and retain characteristics of 3D architecture and shape [39]. To quantitatively measure the effect of liver ECM on metastatic CRC growth, we sectioned the acellular livers into circular discs that were then confined in a 96-well plate for screening (Fig. 3A). It has been shown HCT116 is the most efficient line to metastasize to liver in different animal models [35, 40, 41]. Therefore, we chose to seed HCT116-H2BGFP cells on liver ECM discs and imaged longitudinally using our HCS platform. We imported the segmented cell coordinates and disc images (per well) into a MATLAB script to co-register the disc and cell positions and exclude off-disc cells from the calculations (Fig. 3B). This allowed us to separate cells grown on the disc with those that settled on the background well plate. We used CellPD to calculate the growth rate of cells grown on the discs under different oxygen concentrations. Our results showed that the growth kinetics on liver ECM discs are markedly different from those measured on plastic cell culture plates (Fig. 3C). Interestingly, growth on liver ECM makes the HCT116-H2BGFP cells less sensitive to oxaliplatin treatment under hypoxia, but not under normoxia (Fig. 3D).

Discussion

Metastatic growth in distant organ sites is one of the most challenging areas in cancer treatment. Metastasis is a multi-step process with many studies focusing on molecular changes driving metastatic progression. However, no new gene mutations or amplifications have been clearly linked to metastasis in CRC thus far. The idea that tumor cells “seed” and grow in permissive “soil” was first suggested by Stephen Paget in 1889 [42]. Although Paget’s ideas remain relevant today, many of the underlying mechanisms that explain his observations are poorly understood. Key problems in metastasis remain unsolved, including the organ microenvironment’s role in seeding, survival, and sustained metastatic growth, and its relations with patient outcome. The TME is heterogenous in nature, but traditional biological assays often only examine a single environmental factor at a time, which is not representative of the biology. Our quantitative high-content imaging approach illuminates the dynamic interactions between cancer cells and

treatment response to oxaliplatin under a multiplicity of environmental perturbations, which would be difficult to tune or modulate *in vivo*.

A major determinant of sensitivity to oxaliplatin in CRC cells lines is the p53–p21 pathway [43]. HCT116, a p53 wild type cell line, is strongly inhibited by oxaliplatin treatment, whereas HT29 cells harboring a p53 mutation are less sensitive to this treatment [44, 45]. Our results showing a reduced growth rate of oxaliplatin-treated HCT116 cells under hypoxia, which is not evident in HT29, supports further examination into whether hypoxia alters oxaliplatin-induced p53 activation in CRC cells. It is also interesting to note that HCT116 and HT29 cells are MSI and MSS (microsatellite stable), respectively [44, 45]. It has been shown that moderate hypoxia downregulates DNA mismatch repair (MMR) genes in a HIF-dependent manner, while severe hypoxia can lead to transcriptional repression in a HIF-independent manner [16]. Whether hypoxia-induced down-regulation of MMR genes contributes to the differential oxaliplatin IC_{50} observed in our studies deserves further investigation.

Previous research has shown that the increased metastatic potential of HCT116 cells could be due to enhanced ECM adhesion and haptotaxis [41]. Our data suggests that a stiffer liver metastases (LM) microenvironment may also contribute to the aggressive phenotype of HCT116 cells. Specifically, we found that HCT116 cells grew faster on the 2 kPa stiffness, a physical environment more similar to LM, than on the 0.2 kPa stiffness, and this was not observed in the less aggressive HT29 cell line. Our results also reveal a liver ECM-driven effect that attenuates oxaliplatin-induced HCT116 growth inhibition under hypoxia conditions, which is not evident in LM-mimicking stiffness (2 kPa softwell). This finding may suggest a stiffness-independent crosstalk between liver ECM and hypoxia signaling.

When we examined the combinatorial impact of oxaliplatin treatment and stiffness, we observed a difference in growth rate in the HT29 cells that was not evident in the HCT116 cells. We found LM-mimicking stiffness (2 kPa) increased the relative growth rate of low-dose oxaliplatin-treated HT29 cells under 1% oxygen concentration compared to primary tumor-mimicking stiffness (0.2 kPa). Increased matrix stiffness has been shown to increase stemness characteristics and result in oxaliplatin-resistance through Akt/mTOR pathway [46]. mTOR can also be affected by hypoxia and mediate additional changes in translation [47]. The multifaced interaction between hypoxia, stiffness, and drug resistance warrants further investigation. An advantage of HCS is the ability to assay complex cellular phenotypes in this multiplexed fashion [32]. Understanding multiple microenvironmental interactions is key to developing therapeutic microenvironmental manipulations in liver and other metastatic sites for controlling metastatic tumor growth.

Our CRC imaging dataset has the potential for extensive reuse in multicellular systems biology. Converting quantitative measurements into cell phenotype parameters with CellPD facilitates data sharing and implementation into dynamical computational models. Several computational models have been developed to investigate the dynamics of more invasive phenotypes driven by oxygen-limited environments, as well as the feedback between multicellular cancer systems and the chemical / biophysical microenvironment [48-51]. The impact of ECM has also been included to simulate tumor-associated angiogenesis [52, 53]. Such simulation investigations have yielded substantial insights on the multicellular dynamics of cancer. However, future advances will require high-quality data sets that can be used to formulate single-cell biological hypotheses (simulated cell “rules”), simulate the emergent multicellular behavior, and validate by comparison with imaging and other data [30].

Recent work, using generic tumor cell phenotypic parameters, showed that relatively simple hypotheses on tumor-stromal mechanobiologic feedbacks can lead to complex emergent behaviors in liver metastases including tumor dormancy [54]. The dataset presented in this paper could extend such studies both by providing refined phenotypic parameters and by motivating improved biological hypotheses. In particular, this dataset's measurements on how proliferation varies with tissue stiffness and oxygenation could improve constitutive hypotheses to simulate populations of tumor cells growing in dynamical, heterogeneous conditions. Because we also measured therapeutic response in multiple cell lines in varied TME conditions, those same frameworks could be used to assess evolutionary processes that drive therapeutic resistance in the specific context of the liver parenchyma.

High-throughput quantitative imaging datasets may bridge the gap between traditional biology and computational modeling to enable a systematic investigation of multiple linked microenvironmental factors contributing to CRC metastatic growth and potential therapeutic strategies. The cell phenotype parameters generated from our HCS platform will help build experimentally-driven computational models of metastatic colon cancer cell growth as a function of microenvironment conditions in the liver parenchyma. We can then use these models of metastatic tumor growth to probe the relationships between growth dynamics and heterogeneous microenvironments to facilitate a deeper understanding of complex metastatic processes, and to develop new hypotheses and possible therapeutic interventions. We also envision that multifactorial datasets (including this one) will serve as gold standard data to help drive refinements in dynamical simulation model calibration and validation protocols.

Methods

Cell culture and reagents

The human colorectal cell lines HCT116 and HT29 were acquired from ATCC and cultured in McCoy's 5A medium supplemented with 10% fetal bovine serum (Gemini) and 1% penicillin/streptomycin (Gemini). Caco2 cells were acquired from ATCC and maintained in EMEM supplemented with 10% fetal bovine serum and 1% penicillin/streptomycin. For live cell imaging, HCT116-H2BGFP and HT29-H2BGFP were created by transducing HCT116 and HT29 with LentiBrite Histone H2B-GFP lentivirus (Millipore #17-10229). A positive GFP cell population was selected by a fluorescence-activated cell sorter (FACS). Cell lines were authenticated by a professional authentication service (University of Arizona Genetic Core) and routinely tested for mycoplasma contamination using MycoAlert (Lonza #LT07-518). Hypoxia experiments were carried out in a hypoxia workstation (Biospherix) with separate chambers that allow for precise control over oxygen culture conditions (0.1% - 1% O_2).

Liver ECM disc preparation

Following our published protocol, livers from ferrets age 5-6 weeks were harvested and decellularized using a detergent of deionized water, 1% triton X-100 and 0.1% ammonium hydroxide for three days. The spatial arrangement of collagens I, III and IV, laminin and fibronectin is similar to fresh human liver tissue [39]. Decellularized livers were embedded in OCT for frozen sectioning. The tissue was then sectioned into circular discs with a diameter of 6 mm. The discs were confined in 96 well CellCarrier plates with PBS to prevent tissue dehydration. Prior to CRC cell seeding, discs were washed 3 more times with PBS and then pre-conditioned with culture medium at 37°C for 60 minutes.

Image acquisition and analysis

Endpoint growth rate experiments of HCT116, HT29 and Caco2 were carried out in 96 well CellCarrier plates (PerkinElmer #6005558) at an initial cell seeding of 1500, 4000, and 2000 cells per well, respectively. One day after seeding, cells were treated with the indicated dilutions of oxaliplatin (Selleck Chemicals #S1224). At the stated time points, images were acquired on an Operetta High Content Screening (HCS) System (PerkinElmer #HH12000000) equipped with environmental controls (37°C, 5% CO_2). 30 minutes prior to imaging, cells were stained with 5 µg/ml of Hoechst 33342 (Invitrogen #H21492) and 5 µg/ml of propidium iodine (Invitrogen #P1304MP) to determine live or dead cells, respectively. For live cell experiments, cells were seeded on 0.2 kPa or 2 kPa softwell (Matrigen) or CellCarrier plates in the presence or absence of liver ECM disc. Images were taken on the Operetta HCS in confocal mode using the z-stack function. For all experiments, image analysis was performed using the Harmony 3.5.2 software (PerkinElmer #HH17000001). Cells were identified and segmented at the nuclear level to determine live and dead cell counts over time as described previously [32].

Determination of IC_{50}

The IC_{50} value was determined for each experiment by estimating the oxaliplatin concentration at which the growth rate was 50% of the untreated value via linear interpolation on a log-concentration scale.

Register cells on liver ECM discs

A two-step process was used to separate cells seeded on the disc and on the background well plate. We first segmented the image of the well to on- and off-disc regions and then co-registered the cell locations with the disc region.

Segmenting the disc

Given the local variance at the off-disc region is lower compared to the disc regions, we employed a standard deviation (STD) filter followed by a median filter to find the main structure of the disc and then applied a series of morphological operations to include the small details and trim the noisy non-disc regions close to the borders. We also imaged empty wells to make a light profile for the images and then compared this profile with the images of the wells to add some candidate on-disc pixels before applying morphological operations. We ran the disc segmentation by sweeping over the parameters that control the segmentation and manually chose the best segmentation. The three main parameters used were 1) the kernel size for the STD filtering, 2) the threshold for marking a pixel as a candidate on-disc pixel, and 3) the size of structural elements used for morphological operations.

Co-registering the cell locations with disc

Cell segmentation and the cell's center coordinates were acquired from the Harmony 3.5.2 software. The center of the cell was overlayed with the segmented mask region. By iterating over all the cells, we separated the cells based on a location on or off the disc.

Statistical Analysis

Figure 1. 1B: Increasing or decreasing growth rate across O_2 levels was tested using a two-sided sign test across all same-experiment increasing O_2 levels. This nonparametric procedure is insensitive to cross-experiment measurement variation, and employs only the order of O_2 levels, and not their specific values. The tests were sufficiently powered to detect instances where all data trended in a single direction at a significance level of p=0.05 (Caco2: 6 comparisons, HT29: 7 comparisons, HCT116: 8 comparisons). This criterion was met only for HCT116. Sign tests were performed in *R* using the *SIGN.test* function in the *BSDA* package (v1.2.0). 1D: We tested

for IC_{50} differences between hypoxia (1% or 0.1% O_2) and normoxia (21% O_2) using posterior estimates from an empirical Bayesian model (*brms* package v2.13.5 under R v4.0.2). The model included effects for each hypoxia comparison to normoxia for each cell line. Weakly informative priors were used both for IC_{50} differences (Gaussian) and noise level (Cauchy), each scaled loosely to the data. Reported credible intervals are symmetric 95% intervals of the IC_{50} difference posterior distributions.
Figure 2. We quantified growth rate differences (absolute and relative) from posterior distributions of empirical Bayesian models. Models included categorical population (fixed) effects for cell type (HCT116, HT29) and plate type (Plastic, Softwell 0.2 kPa, Softwell 2 kPa), and a group (random) effect for experiment date (consisting of one plate for each). Residual Gaussian errors were grouped by plate type, each having its own variance estimate. Priors on growth rates (absolute and relative), inter-plate variance, and residual variance were all Cauchy distributed, with zero mean and width order-of-magnitude empirically derived from the data. Estimates of growth rates and their differences are reported as posterior mean and 95% credible (posterior) intervals.
Figure 3. Reported p-values are computed from two-sided Welch's t-tests.

Data Availability

The data sets supporting the results of this article are available in *GigaScience* GigaDB database [55].

List of abbreviations

CRC: colorectal cancer
ECM: extracellular matrix
TME: tumor microenvironment
LM: liver metastasis
HCS: high content screening
MMR: mismatch repair

Funding

This work was supported by an NIH/NCI R01 Provocative Questions (PQ) Grant, CA180149 (awarded to D.B.A., A.A., and S.S.).

Competing interests

The authors declare no competing interests.

Author contributions

C.T.C. and R.L. conducted experiments and analyzed data. A.G. and P.M. wrote MATLAB script to co-register cell locations with disc. E.F.J. and P.M. wrote CellPD to calculate cell growth rate. D.R. performed the statistical analyses. D.V., M.B., S.S., and A.A. generated liver ECM discs. C.T.C., D.B.A., D.R., P.M. and S.M.M. wrote the manuscript and conceptualized the framework for this research. All authors helped edit the manuscript.

Acknowledgement

This work was funded by a National Cancer Institute Grant R01 CA180149. Additional support was provided by the USC Medical Faculty Women's Association (awarded to S.M.M.). We thank A. Skardal (Ohio State University) and R. Sun for technical support and N. Matasci and T. Gerhart for script maintenance. We would also like to express our deepest gratitude to our philanthropic supporters: the Stephenson family, Emmet, Toni and Tessa, for their donation of the Operetta HCS platform, which was instrumental in carrying out this work.

Figure titles and legends

Fig. 1. The impact of oxygen on CRC growth and treatment response to oxaliplatin. a Caco2, HT29, and HCT116 cells were cultured in 0.1%, 1% or 21% oxygen concentration. Cell counts were measured at several time points using Operetta high-content screening platform. **b** Growth rate of Caco2, HT29, and HCT116 cells in 0.1%, 1% or 21% oxygen concentration was determined by CellPD. A two-sided sign test was used to detect instances where all data trended in a single direction. **c** Relative growth rate of Caco2, HT29, and HCT116 cells treated with 0.167, 0.5, 1.67, 5 μM oxaliplatin. **d** Oxaliplatin IC_{50} changes in 0.1, 1 or 21% oxygen environment.

Fig. 2. The influence of stiffness on CRC growth and treatment response to oxaliplatin. a HT29-H2BGFP and HCT116-H2BGFP cells were cultured on 0.2 kPa or 2 kPa gel (softwell) or plastic (CellCarrier) plates in 1% or 21% oxygen concentration for 72 hours. Cell counts were measured at several time points by Operetta high-content screening platform, and the growth rate was determined by CellPD. **b** Relative growth rate of HT29-H2BGFP and HCT116-H2BGFP cells in response to 0.5 or 5 μM oxaliplatin treatment in 1% or 21% oxygen concentration.

Fig.3. The effect of liver ECM on the growth of CRC cells and treatment response to oxaliplatin. a Liver ECM discs were sectioned from acellular liver and seeded with HCT116-H2BGFP cells. **b** Snapshots of the disc segmentation process: (1) applying STD filtering and median filtering to the well; (2) applying dilation-reconstruction morphological operations, thresholding and drawing the segmented region over the original image; (3) separating the cells to on-disc and off-disc sets based on the cell location. The on-disc cells were colored green and off-disc cells were red. **c** HCT116-H2BGFP cells were cultured on liver ECM disc or monolayer under 1% or 21% oxygen concentration for 72 hours. Cell counts were measured at several time points by Operetta HCS platform, and the growth rate was determined by CellPD. **d** Relative growth rate of HCT116-H2BGFP cells in response to 0.5 or 5 μM oxaliplatin treatment under 1% or 21 % oxygen concentration. $^*P < 0.05$

References

1. Siegel RL, Miller KD and Jemal A. Cancer statistics, 2019. CA Cancer J Clin. 2019;69 1:7-34. doi:10.3322/caac.21551.
2. Sanchez-Gundin J, Fernandez-Carballido AM, Martinez-Valdivieso L, Barreda-Hernandez D and Torres-Suarez AI. New Trends in the Therapeutic Approach to Metastatic Colorectal Cancer. Int J Med Sci. 2018;15 7:659-65. doi:10.7150/ijms.24453.
3. Lee RM, Cardona K and Russell MC. Historical perspective: Two decades of progress in treating metastatic colorectal cancer. J Surg Oncol. 2019;119 5:549-63. doi:10.1002/jso.25431.
4. Willyard C. Cancer therapy: an evolved approach. Nature. 2016;532 7598:166-8. doi:10.1038/532166a.
5. Tan Q, Saggar JK, Yu M, Wang M and Tannock IF. Mechanisms of Drug Resistance Related to the Microenvironment of Solid Tumors and Possible Strategies to Inhibit Them. Cancer J. 2015;21 4:254-62. doi:10.1097/ppo.0000000000000131.
6. Mumenthaler SM, Foo J, Choi NC, Heise N, Leder K, Agus DB, et al. The Impact of Microenvironmental Heterogeneity on the Evolution of Drug Resistance in Cancer Cells. Cancer Inform. 2015;14 Suppl 4:19-31. doi:10.4137/cin.s19338.
7. Straussman R, Morikawa T, Shee K, Barzily-Rokni M, Qian ZR, Du J, et al. Tumour micro-environment elicits innate resistance to RAF inhibitors through HGF secretion. Nature. 2012;487 7408:500-4. doi:10.1038/nature11183.

8. Zarour LR, Anand S, Billingsley KG, Bisson WH, Cercek A, Clarke MF, et al. Colorectal Cancer Liver Metastasis: Evolving Paradigms and Future Directions. Cell Mol Gastroenterol Hepatol. 2017;3 2:163-73. doi:10.1016/j.jcmgh.2017.01.006.
9. Burke D and Allen-Mersh TG. Colorectal liver metastases. Postgrad Med J. 1996;72 850:464-9. doi:10.1136/pgmj.72.850.464.
10. Bocuk D, Wolff A, Krause P, Salinas G, Bleckmann A, Hackl C, et al. The adaptation of colorectal cancer cells when forming metastases in the liver: expression of associated genes and pathways in a mouse model. BMC Cancer. 2017;17 1:342. doi:10.1186/s12885-017-3342-1.
11. Catarinella M, Monestiroli A, Escobar G, Fiocchi A, Tran NL, Aiolfi R, et al. IFNalpha gene/cell therapy curbs colorectal cancer colonization of the liver by acting on the hepatic microenvironment. EMBO Mol Med. 2016;8 2:155-70. doi:10.15252/emmm.201505395.
12. Van den Eynden GG, Majeed AW, Illemann M, Vermeulen PB, Bird NC, Hoyer-Hansen G, et al. The multifaceted role of the microenvironment in liver metastasis: biology and clinical implications. Cancer research. 2013;73 7:2031-43. doi:10.1158/0008-5472.can-12-3931.
13. Quail DF and Joyce JA. Microenvironmental regulation of tumor progression and metastasis. Nature medicine. 2013;19 11:1423-37. doi:10.1038/nm.3394.
14. Gelman S and Mushlin PS. Catecholamine-induced changes in the splanchnic circulation affecting systemic hemodynamics. Anesthesiology. 2004;100 2:434-9. doi:10.1097/00000542-200402000-00036.
15. Brown JM and Wilson WR. Exploiting tumour hypoxia in cancer treatment. Nature reviews Cancer. 2004;4 6:437-47. doi:10.1038/nrc1367.
16. Scanlon SE and Glazer PM. Multifaceted control of DNA repair pathways by the hypoxic tumor microenvironment. DNA repair. 2015;32:180-9. doi:10.1016/j.dnarep.2015.04.030.
17. Boland CR and Goel A. Microsatellite instability in colorectal cancer. Gastroenterology. 2010;138 6:2073-87.e3. doi:10.1053/j.gastro.2009.12.064.
18. Pickup MW, Mouw JK and Weaver VM. The extracellular matrix modulates the hallmarks of cancer. EMBO Rep. 2014;15 12:1243-53. doi:10.15252/embr.201439246.
19. Cox TR and Erler JT. Remodeling and homeostasis of the extracellular matrix: implications for fibrotic diseases and cancer. Disease models & mechanisms. 2011;4 2:165-78. doi:10.1242/dmm.004077.
20. Mohammadi H and Sahai E. Mechanisms and impact of altered tumour mechanics. Nature Cell Biology. 2018;20 7:766-74. doi:10.1038/s41556-018-0131-2.
21. Butcher DT, Alliston T and Weaver VM. A tense situation: forcing tumour progression. Nature reviews Cancer. 2009;9 2:108-22. doi:10.1038/nrc2544.
22. Nagelkerke A, Bussink J, Rowan AE and Span PN. The mechanical microenvironment in cancer: How physics affects tumours. Semin Cancer Biol. 2015;35:62-70. doi:10.1016/j.semcancer.2015.09.001.
23. Laklai H, Miroshnikova YA, Pickup MW, Collisson EA, Kim GE, Barrett AS, et al. Genotype tunes pancreatic ductal adenocarcinoma tissue tension to induce matricellular fibrosis and tumor progression. Nature Medicine. 2016;22 5:497-505. doi:10.1038/nm.4082.
24. Colpaert CG, Vermeulen PB, Fox SB, Harris AL, Dirix LY and Van Marck EA. The presence of a fibrotic focus in invasive breast carcinoma correlates with the expression of carbonic anhydrase IX and is a marker of hypoxia and poor prognosis. Breast Cancer Research and treatment. 2003;81 2:137-47. doi:10.1023/a:1025702330207.
25. Shen Y, Wang X, Lu J, Salfenmoser M, Wirsik NM, Schleussner N, et al. Reduction of Liver Metastasis Stiffness Improves Response to Bevacizumab in Metastatic Colorectal Cancer. Cancer cell. 2020;37 6:800-17.e7. doi:10.1016/j.ccell.2020.05.005.

26. Mook OR, van Marle J, Jonges R, Vreeling-Sindelarova H, Frederiks WM and Van Noorden CJ. Interactions between colon cancer cells and hepatocytes in rats in relation to metastasis. Journal of cellular and molecular medicine. 2008;12 5b:2052-61. doi:10.1111/j.1582-4934.2008.00242.x.
27. Kaminska K, Szczylik C, Bielecka ZF, Bartnik E, Porta C, Lian F, et al. The role of the cell-cell interactions in cancer progression. Journal of cellular and molecular medicine. 2015;19 2:283-96. doi:10.1111/jcmm.12408.
28. Metzcar J, Wang Y, Heiland R and Macklin P. A Review of Cell-Based Computational Modeling in Cancer Biology. JCO Clin Cancer Inform. 2019;3:1-13. doi:10.1200/cci.18.00069.
29. Macklin P, Frieboes HB, Sparks JL, Ghaffarizadeh A, Friedman SH, Juarez EF, et al. Progress Towards Computational 3-D Multicellular Systems Biology. Advances in experimental medicine and biology. 2016;936:225-46. doi:10.1007/978-3-319-42023-3_12.
30. Macklin P. Key challenges facing data-driven multicellular systems biology. Gigascience. 2019;8 10 doi:10.1093/gigascience/giz127.
31. Zock JM. Applications of high content screening in life science research. Comb Chem High Throughput Screen. 2009;12 9:870-76.
32. Garvey CM, Spiller E, Lindsay D, Chiang CT, Choi NC, Agus DB, et al. A high-content image-based method for quantitatively studying context-dependent cell population dynamics. Sci Rep. 2016;6:29752. doi:10.1038/srep29752.
33. Chiang CT, Demetriou AN, Ung N, Choudhury N, Ghaffarian K, Ruderman DL, et al. mTORC2 contributes to the metabolic reprogramming in EGFR tyrosine-kinase inhibitor resistant cells in non-small cell lung cancer. Cancer Lett. 2018;434:152-9. doi:10.1016/j.canlet.2018.07.025.
34. Sullivan R, Pare GC, Frederiksen LJ, Semenza GL and Graham CH. Hypoxia-induced resistance to anticancer drugs is associated with decreased senescence and requires hypoxia-inducible factor-1 activity. Mol Cancer Ther. 2008;7 7:1961-73. doi:10.1158/1535-7163.mct-08-0198.
35. Hamada K, Monnai M, Kawai K, Nishime C, Kito C, Miyazaki N, et al. Liver metastasis models of colon cancer for evaluation of drug efficacy using NOD/Shi-scid IL2Rgammanull (NOG) mice. Int J Oncol. 2008;32 1:153-9.
36. Juarez EF, Lau R, Friedman SH, Ghaffarizadeh A, Jonckheere E, Agus DB, et al. Quantifying differences in cell line population dynamics using CellPD. BMC Syst Biol. 2016;10 1:92. doi:10.1186/s12918-016-0337-5.
37. Alcindor T and Beauger N. Oxaliplatin: a review in the era of molecularly targeted therapy. Current oncology (Toronto, Ont). 2011;18 1:18-25. doi:10.3747/co.v18i1.708.
38. Erler JT and Giaccia AJ. Lysyl oxidase mediates hypoxic control of metastasis. Cancer research. 2006;66 21:10238-41. doi:10.1158/0008-5472.can-06-3197.
39. Baptista PM, Siddiqui MM, Lozier G, Rodriguez SR, Atala A and Soker S. The use of whole organ decellularization for the generation of a vascularized liver organoid. Hepatology. 2011;53 2:604-17. doi:10.1002/hep.24067.
40. Xu Y, Zhang L, Wang Q and Zheng M. Comparison of Different Colorectal Cancer With Liver Metastases Models Using Six Colorectal Cancer Cell Lines. Pathology oncology research : POR. 2020;26 4:2177-83. doi:10.1007/s12253-020-00805-3.
41. Ishizu K, Sunose N, Yamazaki K, Tsuruo T, Sadahiro S, Makuuchi H, et al. Development and characterization of a model of liver metastasis using human colon cancer HCT-116 cells. Biological & pharmaceutical bulletin. 2007;30 9:1779-83. doi:10.1248/bpb.30.1779.
42. Psaila B and Lyden D. The metastatic niche: adapting the foreign soil. Nature Reviews Cancer. 2009;9 4:285-93. doi:10.1038/nrc2621.

43. Toscano F, Parmentier B, Fajoui ZE, Estornes Y, Chayvialle JA, Saurin JC, et al. p53 dependent and independent sensitivity to oxaliplatin of colon cancer cells. Biochemical pharmacology. 2007;74 3:392-406. doi:10.1016/j.bcp.2007.05.001.
44. Berg KCG, Eide PW, Eilertsen IA, Johannessen B, Bruun J, Danielsen SA, et al. Multi-omics of 34 colorectal cancer cell lines - a resource for biomedical studies. Molecular cancer. 2017;16 1:116. doi:10.1186/s12943-017-0691-y.
45. Ahmed D, Eide PW, Eilertsen IA, Danielsen SA, Eknæs M, Hektoen M, et al. Epigenetic and genetic features of 24 colon cancer cell lines. Oncogenesis. 2013;2 9:e71. doi:10.1038/oncsis.2013.35.
46. You Y, Zheng Q, Dong Y, Xie X, Wang Y, Wu S, et al. Matrix stiffness-mediated effects on stemness characteristics occurring in HCC cells. Oncotarget. 2016;7 22:32221-31. doi:10.18632/oncotarget.8515.
47. Wouters BG and Koritzinsky M. Hypoxia signalling through mTOR and the unfolded protein response in cancer. Nature reviews Cancer. 2008;8 11:851-64. doi:10.1038/nrc2501.
48. Ghaffarizadeh A, Heiland R, Friedman SH, Mumenthaler SM and Macklin P. PhysiCell: An open source physics-based cell simulator for 3-D multicellular systems. PLoS Comput Biol. 2018;14 2:e1005991. doi:10.1371/journal.pcbi.1005991.
49. Anderson AR, Rejniak KA, Gerlee P and Quaranta V. Microenvironment driven invasion: a multiscale multimodel investigation. J Math Biol. 2009;58 4-5:579-624. doi:10.1007/s00285-008-0210-2.
50. Gatenby RA, Smallbone K, Maini PK, Rose F, Averill J, Nagle RB, et al. Cellular adaptations to hypoxia and acidosis during somatic evolution of breast cancer. Br J Cancer. 2007;97 5:646-53. doi:10.1038/sj.bjc.6603922.
51. Drasdo D and Hohme S. A single-cell-based model of tumor growth in vitro: monolayers and spheroids. Phys Biol. 2005;2 3:133-47. doi:10.1088/1478-3975/2/3/001.
52. Cai Y, Xu S, Wu J and Long Q. Coupled modelling of tumour angiogenesis, tumour growth and blood perfusion. J Theor Biol. 2011;279 1:90-101. doi:10.1016/j.jtbi.2011.02.017.
53. Bauer AL, Jackson TL and Jiang Y. A cell-based model exhibiting branching and anastomosis during tumor-induced angiogenesis. Biophys J. 2007;92 9:3105-21. doi:10.1529/biophysj.106.101501.
54. Wang, Y., Brodin, E., Nishii, K. et al. Impact of tumor-parenchyma biomechanics on liver metastatic progression: a multi-model approach. Sci Rep, 2021; 11 1710. https://doi.org/10.1038/s41598-020-78780-7.
55. Chiang CT, Lau R, Ghaffarizadeh A, Brovold M, Vyas D, Juárez EF, et al. Supporting data for "High throughput microscopy reveals the impact of multifactorial environmental perturbations on colorectal cancer cell growth". *GigaScience* Database. 2021; . http://dx.doi.org/10.5524/100849

Figure

Click here to access/download;Figure;Figures_Gigascience_revision_v3.ppt

Fig. 1. The impact of oxygen on CRC growth and treatment response to oxaliplatin.

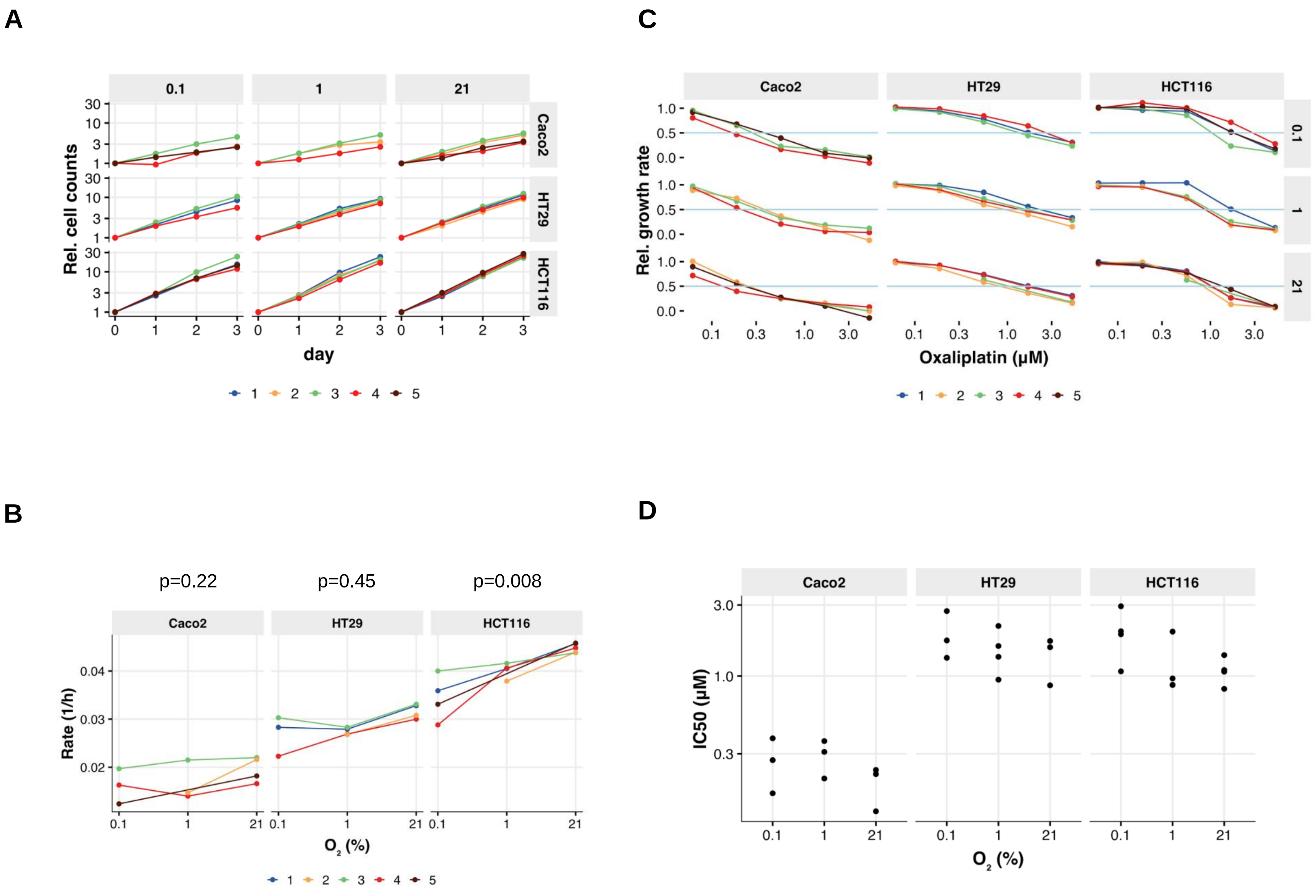

Fig. 2. The influence of stiffness on CRC growth and treatment response to oxaliplatin.

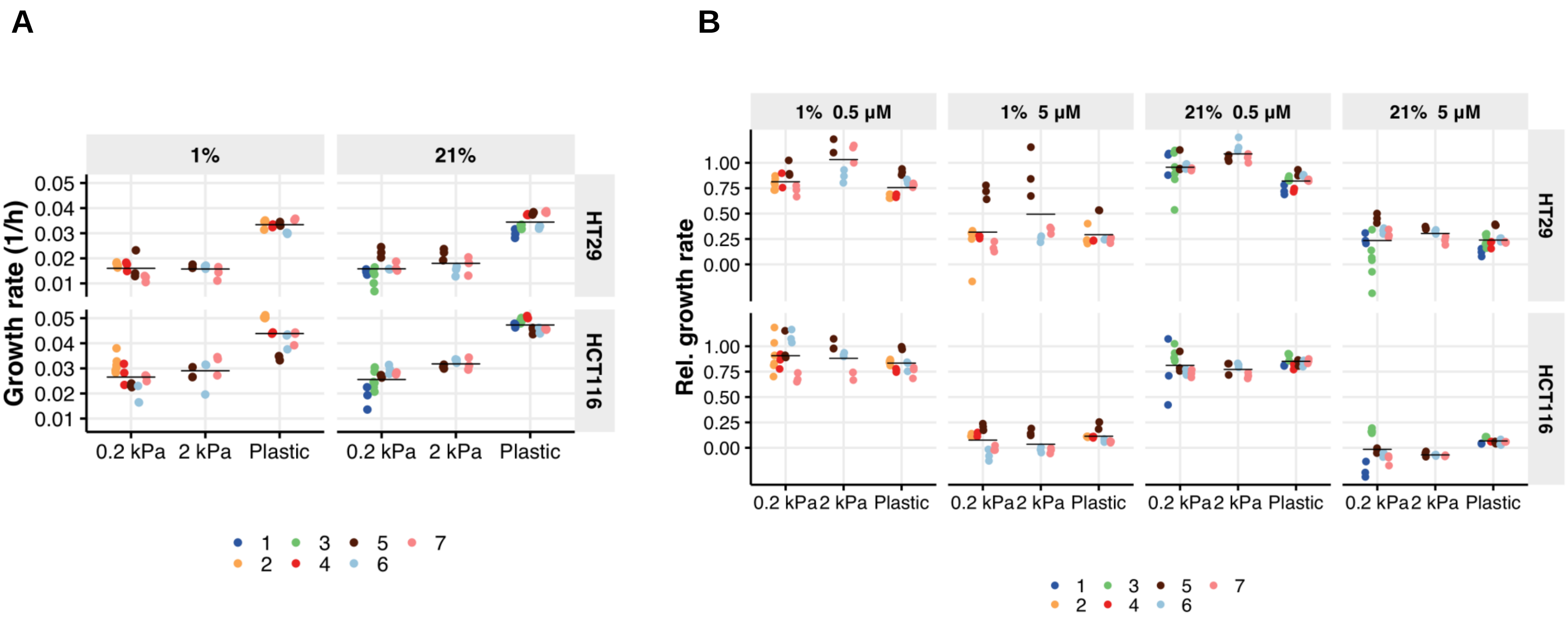

Fig. 3. The effect of liver ECM on the growth of CRC cells and treatment response to oxaliplatin.

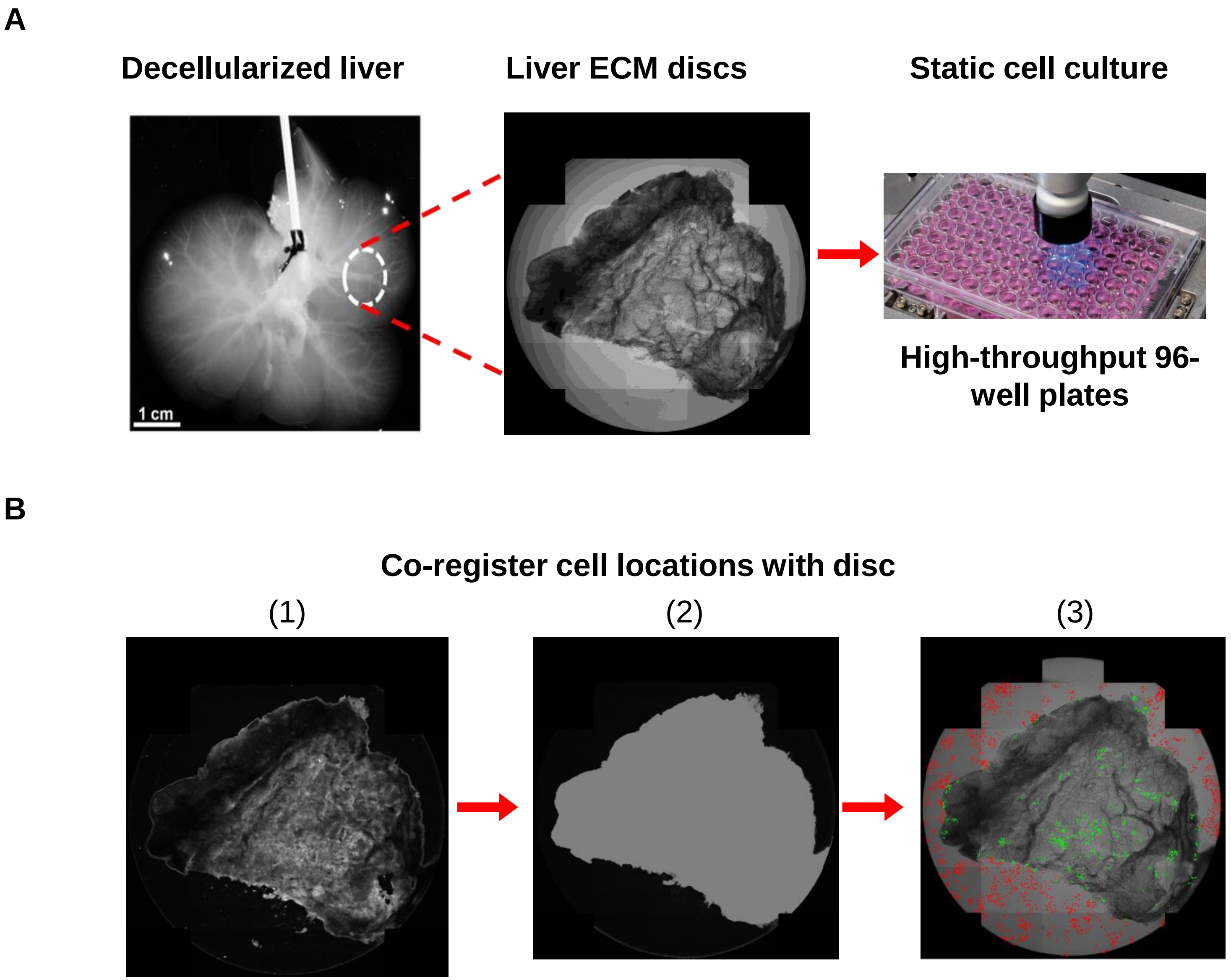

Fig. 3. The effect of liver ECM on the growth of CRC cells and treatment response to oxaliplatin.

C

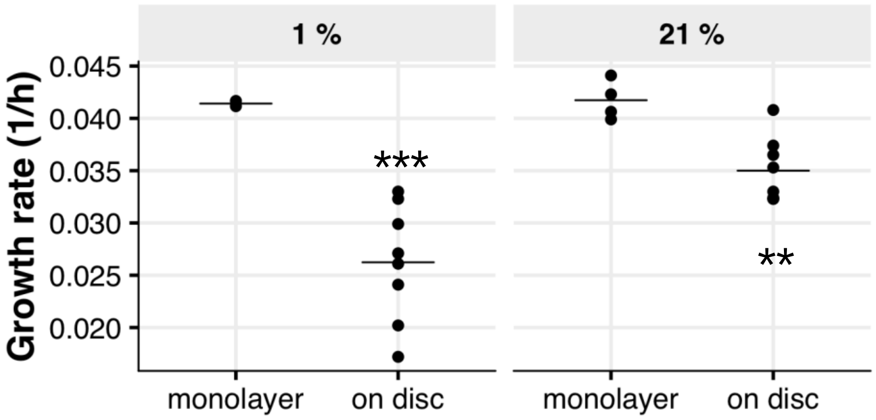

D

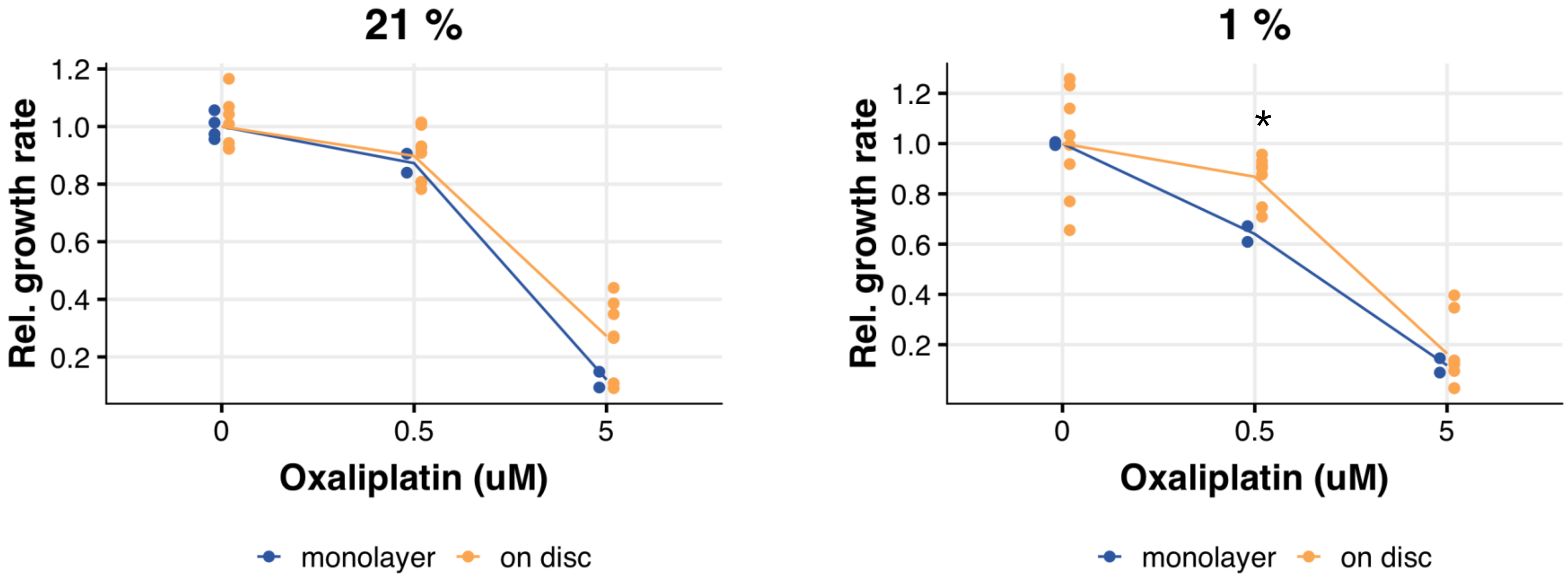

Click here to access/download
Supplementary Material
SupplementaryFigures_Gigascience_v9.ppt

Personal Cover

Click here to access/download;Personal Cover;CoverLetter_Revision_final.pdf

Lawrence J. Ellison Institute for Transformative Medicine of USC

November 20, 2020

Dear Hongling Zhou,

Thank you for your letter on June 29th 2020, which included the constructive comments made by three independent reviewers. On behalf of all of my co-authors, I am pleased to submit a revised version of GIGA-D-20-00103 entitled, "High throughput microscopy reveals the impact of multifactorial environmental perturbations on colorectal cancer cell growth".

We have uploaded a copy of the revised manuscript and a point-by-point response to the reviewers' concerns. Based on important feedback from the reviewers, we incorporated additional background information and discussion points to improve the significance and clarity of the manuscript. Additionally, we performed 3 biological replicate experiments to address reviewers' comments, and the supporting data of these experiments, including raw images, are transferring to the FTP server (ftp://user55@parrot.genomics.cn). We also re-ran the IC50 analyses in Fig. 1 using the newer versions of R packages so everything was run on the same version, and the results are unchanged.

We truly appreciate the valuable feedback from the reviewers and feel that the manuscript is much stronger as a result. Thank you for the opportunity to submit a revised manuscript. If you have any further questions, please don't hesitate to contact me.

Sincerely,

Shannon Mumenthaler

Shannon Mumenthaler, Ph.D.
Assistant Professor of Medicine
Department of Medicine

University of Southern California
2250 Alcazar Street, CSC 240, Mail Code 9075, Los Angeles, California 90033 • Tel: 323 442 2529 • Fax: 323 442 2764 • smumenth@usc.edu
