# Supplementary material for: High-throughput microscopy reveals the impact of multifactorial environmental perturbations on colorectal cancer cell growth
Source: Gigascience. 2021 Apr 19;10(4):giab026. doi: 10.1093/gigascience/giab026 (PMC8054261; doi:10.1093/gigascience/giab026)
Supplement: giab026_Response_to_Reviewer_Comments_Original_Submission [file giab026_response_to_reviewer_comments_original_submission.pdf]

# Author's Response To Reviewer Comments

Close

Summary of Response to Reviewers

General Comments: We greatly appreciate the comments of the reviewers and find that their suggestions have made this a much stronger manuscript. We have addressed each point in a revised manuscript and have documented the major edits under "Actions taken."

Response to reviewer #1

This excellent Research Article utilises a quantitative imaging approach to elucidate the effects of hypoxia, chemotherapeutics, and extracellular matrix stiffness in an in vitro model of colorectal cancer. Three different colorectal cancer cell lines were used in this study and growth rates were quantified using an Operetta High Content Imaging system. I was particularly intrigued by the novel use of decellularized liver scaffolds as a means of assessing the role of tissue architecture on colorectal tumor cell growth. The image analysis reported in this study highlights that growth kinetics of colorectal tumour cells on decellularized liver scaffolds is "markedly different" from plastic cell culture plates, and that HCT116-H2BGFP cells cultured on liver ECM were less sensitive to oxaliplatin treatment under hypoxic conditions. From a biomedical perspective, where the tropism of metastatic colorectal cancer cells for liver is well established, I consider this finding especially relevant.

The supporting image data for this study have been submitted to GigaScience. These data are extremely well organised and I thank the authors for providing details of: row, column, field, plane, channel, and time point for each of the 16-bit images used in this study. In addition, the supporting analyses have also been submitted to GigaScience as tabular data, as have the supporting scripts.

I accept this article for publication in GigaScience. Furthermore, there is a clear need for benchmark datasets of this nature, and I fully support the authors' request to include this manuscript in the GigaScience thematic series entitled "Data-Driven Multicellular Systems Biology."

We thank the reviewer for their valuable assessment of our manuscript and appreciate the feedback that was provided.

Response to reviewer #2

The article is simple, straightforward and easy to follow. The ability of high content screening to measure phenotypic changes with alterations to the tumor microenvironment such as hypoxia and matrix stiffness has been studied using 3 different cell lines. In addition to modifying oxygen levels, the authors also perform co-cultures with an interesting acellular liver ECM disk to observe the effect of liver scaffolds in tumor growth patterns.

We thank Reviewer 2 for the positive assessment of our manuscript and the constructive comments for improvement. Due to the coronavirus and university shut-downs, we were unable to perform additional liver ECM disc and animal studies. However, we have carefully addressed the reviewer's additional comments with new softwell experiments and modifications to the text in this revised version of the manuscript and in a point-by-point response below.

While figures 1-2 report changes in multiple cell lines, figure 4 highlights the effect of liver scaffolds on only the most aggressive and metastatic cell line HCT116. Reporting the effect of liver scaffold on the growth of less aggressive and metastatic cell lines and comparing and contrasting the differences would have been more effective at studying ECM based regulation of tumor growth. The authors could also have utilized more fluorescent markers in addition to DAPI to determine the effect of the hypoxia and liver ECM on tumor biology.

We thank the reviewer for pointing this out. Given HCT116 is one of the most efficient CRC cell lines to metastasize to liver in different animal models, we prioritized the measurements of this line in response to the microenvironmental perturbation on the liver ECM discs. We agree with the reviewer that investigating why certain cell lines do not grow well in the liver is a very interesting and important topic to study. While a detailed assessment is beyond the scope of this manuscript; we did perform additional softwell experiments that more closely mimic the tissue stiffness of liver metastases (LM) (2 kPa) to quantify the growth capabilities of HCT116 and HT29 cells. We found HCT116 cells grow faster on the 2 kPa stiffness that is more similar to LM than on the 0.2 kPa stiffness which is more similar to the primary colon tumor, but this was not observed in the less aggressive HT29 cell line. We believe these data provide important insights for future studies to compare the differential adaptation of CRC cells to the liver microenvironment. Interestingly, we also found the LM stiffness has a protective effect against low dose oxaliplatin-induced cytotoxicity of HT29 cells under 1% oxygen concentration; highlighting the multiplexing capabilities of high content screening. We further discuss the potential for incorporation of additional markers for cell phenotyping using high content screening.

Actions taken
Figs. 2A and 2B > Added 3 biological replicate experiments and revised figures with a side-by-side comparison of the growth of HCT116 and HT29 cells and treatment response to oxaliplatin on 0.2 kPa softwell (mimics tissue stiffness of primary colon tumor), 2 kPa softwell (mimics tissue stiffness of LM) and conventional plastic (3 GPa). All additional raw data files have been uploaded to GigaDB.

Results > Added citation Shen et al. (ref25) and mentioned that 0.2 kPa and 2 kPa are close to the stiffness of primary CRC and liver metastases, respectively. Summarized the results of HCT116 and HT29 cells in response to different stiffness.

Results > Added citations Xu et al. (ref40) and Ishizu et al. (ref41). Revised text to point out that HCT116 is the most efficient line to develop liver metastasis in orthotopic and ectopic animal models to support the choice for examining HCT116 in the liver ECM experiments.

Discussion > Added citation Ishizu et al. (ref41). Discussed the stiffer LM microenvironment may also contribute to the aggressive phenotypes of HCT116 cells.

Discussion > Added citations You et al. (ref46) and Wouters et al. (ref47). Discussed the potential use of other markers to further interrogate the relationship between hypoxia and stiffness in drug sensitivity.

Did the authors test any more chemotherapeutic drugs other than oxaliplatin ? And, what about testing combinations of drugs under these culture conditions ?
Were there plans to implant the cell lines grown in co-cultures or normoxic conditions in mice to follow tumor growth ?

We thank the reviewer for the comment and suggestion. We chose oxaliplatin because it is one of the most effective cytotoxic compounds used in the adjuvant and advanced setting of CRC treatment. We agree with the reviewer that testing additional drugs and drug combinations both in vitro and in vivo would be interesting; however, we believe these studies are beyond the scope of this manuscript and best suited to include in a follow-up manuscript.

Actions taken
Results > Added citation Alcindor et al. (ref37). Revised text to emphasize that oxaliplatin is commonly used in the clinical setting.

Fig 3B (what's the purpose of the yellow arrow mark?)

Thank you for making this point. We are sorry that our presentation may have been confusing. The purpose of the arrow was to connect steps (1), (2), (3) to demonstrate how we segment the cells on the discs.

Actions taken
Figures> Labeled steps 1, 2, and 3 in Fig. 3B to avoid confusion.

Response to reviewer #3

The manuscript High throughput microscopy reveals the impact of multifactorial environmental perturbations on colorectal cancer cell growth and shows that HCT116 cell growth is affected by oxygen, substrate stiffness, and liver ECM. This data set could be set for training and testing data driven computational models of cancer and other multicellular systems.
The manuscript is well written however, there are some major issues I have with the manuscript:

We appreciate Reviewer 3's thorough review of our manuscript and valuable comments. We are delighted that the reviewer found our findings very interesting. The reviewer feedback has been instrumental in improving the overall impact of our manuscript. We have addressed each concern in a revised manuscript and have documented the changes in a point-by-point response below.

1. There is no stiffness mention in the background. It is only mentioned briefly in one sentence, but it is essential for your results and it is in the conclusion of the abstract. There should be at least a paragraph about stiffness in the background.

We thank the reviewer for pointing this out and have added a paragraph in the introduction to emphasize the importance of tissue stiffness in the tumor microenvironment and to address the relationship between stiffness and liver metastasis in colorectal cancer.

Actions taken
Introduction > Added citations Cox et al. (ref19), Mohammadi et al. (ref20), Nagelkerke et al. (ref22), Laklai et al. (ref23), Colpaert et al. (ref24), and Shen et al. (ref25). Included text regarding stiffness and its relationship with liver metastasis.

2. Why using 0.2kPa and 3GPa in the experiments? Those two values are not physiological stiffness. It would be useful to do those experiments with physiological values for example the kPa of a tumor and a normal cell and the 3GPa.

We appreciate the reviewer bringing up the physiological stiffness values in cancer. Based on a recent Cancer Cell paper (PMID: 32516590), the stiffness of liver metastases in metastatic colorectal cancer is significantly higher compared with the primary tumor. The stiffness of primary colorectal cancer is close to 0.2 kPa, and the stiffness of liver metastases is around 2 kPa. Therefore, we performed additional experiments to measure the growth and treatment response of colorectal cancer cells in a 2 kPa environment.

Actions taken
Figs. 2A and 2B > Added 3 biological replicate experiments and revised figures with a side-by-side comparison of the growth of HCT116 and HT29 cells and treatment response to oxaliplatin on 0.2 kPa softwell (mimics tissue stiffness of primary colon tumor), 2 kPa softwell (mimics tissue stiffness of LM) and conventional plastic (3 GPa). All additional raw data files have been uploaded to GigaDB.

Results > Added citation Shen et al. (ref25) and mentioned that the stiffness of 0.2 kPa and 2 kPa are close to the stiffness of primary CRC and liver metastasis, respectively. Summarized the results of HCT116 and HT29 cells in response to different stiffness.

3. Discussion needs further editing.

Thank you for making this point. We added discussion points regarding the differential response to hypoxia between HCT116 and HT29 cells and whether this might be due to differences in p53 and MSI status. In addition, we discussed the differential adaptation of CRC cells to the liver microenvironment. We also discussed potential pathway analyses that can be further explored in future mechanistic and modeling studies. We also carefully reviewed to correct for any previous grammatical errors.

Actions taken
Discussion > Added citations Toscano et al. (ref43), Berg et al. (ref44), Ahmed et al. (ref45), and Scanlon et al. (ref16). Discussed the differential response to hypoxia between HCT116 and HT29 cells.

Discussion > Added citations Ishizu et al. (ref41). Discussed the stiffer LM microenvironment may also contribute to the aggressive phenotypes of HCT116 cells.

Discussion > Added citations You et al. (ref46) and Wouters et al. (ref47). Discussed the relationship

between hypoxia and stiffness in drug sensitivity.

Discussion > Added citation Wang et al. (ref54). Discussed how the phenotypic parameters generated from this study could motivate new biological hypotheses.

4. HT-29 are MSS and HCT116 are MSI. There is some literature about hypoxia and MSI which could be discussed and mentioned in the background

This is an excellent suggestion. We have mentioned the relationship between hypoxia and MSI in the introduction and discussed the potential impact of MSI status on the differential results observed between HCT116 and HT29.

Actions taken
Introduction > Added citations Gelman et al. (ref14), Brown et al. (ref15), Scanlon et al. (ref16), and Boland et al. (ref17) to show hypoxia is particularly pervasive in the liver microenvironment and hypoxia could reduce DNA mismatch repair gene expression and enrich for mismatch repair deficient cells.

Discussion > Added citations Toscano et al. (ref43), Berg et al. (ref44), and Ahmed et al. (ref45), and Scanlon et al. (ref16) to highlight that HCT116 and HT29 cells are MSI and MSS, respectively and to discuss the differential response to hypoxia between HCT116 and HT29 cells.

5. Figure 1 to figure 2 needs a better transfer why the author is doing this experiment.

We thank the reviewer for this comment and agree that there is a connection between hypoxia and stiffness that should be mentioned as a transition from Fig.1 to Fig. 2.

Actions taken
Results > Added citations Laklai et al. (ref23) and Erler et al. (ref38) and added text describing how hypoxia can modulate tumor progression through increased ECM crosslinking and stiffening. Also added citation Shen et al. (ref25) to demonstrate that increased stiffness in liver metastases plays a pivotal role in CRC progression.

6. To really say that this data set could be used for training and testing data driven computational models of cancer and other multicellular systems it would need more than 3 cell lines to perform these experiments. Also, some human data would be useful to make this statement.

Thank you to the reviewer for making this point. The purpose of this study is not to train an AI model across many cell types, but rather use this type of data to calibrate mechanistic, dynamical simulation models of individual cell types. Also, the cell lines used in this manuscript (HCT116, HT29, and Caco2) are often used in such multidisciplinary studies. We modified the abstract to avoid confusion and further edited the discussion on these points. We note that the experiments presented in this manuscript provided sufficient data to determine the net cell proliferation rate for each cell line under a broad variety of oxygenation, ECM, and oxaliplatin treatment conditions. These can be directly incorporated into dynamical models, as is planned for follow-up studies. Moreover, each cell line's plotted curves of proliferation versus O2 (Fig. 1b), proliferation versus drug (Fig. 1c), IC50 versus O2 (Fig. 1D), growth versus ECM stiffness and drug (Fig. 2), and cell proliferation on ECM discs (Fig. 3) will be of great benefit to mathematical biologists as they postulate biological hypotheses to drive refined dynamical models.

Actions taken
Abstract > Edited "data-driven computational models of cancer and other multicellular systems" to "data-driven dynamical models of cancer cell lines and therapeutic response in a variety of microenvironmental conditions."

Discussion > Added citation Wang et al. (ref54). Discussed how the phenotypic parameters generated from this study could motivate new biological hypotheses.

Close
