# Supplementary material for: High-throughput microscopy reveals the impact of multifactorial environmental perturbations on colorectal cancer cell growth
Source: Gigascience. 2021 Apr 19;10(4):giab026. doi: 10.1093/gigascience/giab026 (PMC8054261; doi:10.1093/gigascience/giab026)
Supplement: giab026_Reviewer_1_Report_Original_Submission — Chris Armit -- 4/17/2020 Reviewed [file giab026_reviewer_1_report_original_submission.pdf]

Reviewer Report

Title: High throughput microscopy reveals the impact of multifactorial environmental perturbations on colorectal cancer cell growth

Version: Original Submission **Date:** 4/17/2020

Reviewer name: Chris Armit

Reviewer Comments to Author:

This excellent Research Article utilises a quantitative imaging approach to elucidate the effects of hypoxia, chemotherapeutics, and extracellular matrix stiffness in an in vitro model of colorectal cancer. Three different colorectal cancer cell lines were used in this study and growth rates were quantified using an Operetta High Content Imaging system. I was particularly intrigued by the novel use of decellularized liver scaffolds as a means of assessing the role of tissue architecture on colorectal tumor cell growth. The image analysis reported in this study highlights that growth kinetics of colorectal tumour cells on decellularized liver scaffolds is "markedly different" from plastic cell culture plates, and that HCT116-H2BGFP cells cultured on liver ECM were less sensitive to oxaliplatin treatment under hypoxic conditions. From a biomedical perspective, where the tropism of metastatic colorectal cancer cells for liver is well established, I consider this finding especially relevant.
The supporting image data for this study have been submitted to GigaScience. These data are extremely well organised and I thank the authors for providing details of: row, column, field, plane, channel, and time point for each of the 16-bit images used in this study. In addition, the supporting analyses have also been submitted to GigaScience as tabular data, as have the supporting scripts.
I accept this article for publication in GigaScience. Furthermore, there is a clear need for benchmark datasets of this nature, and I fully support the authors' request to include this manuscript in the GigaScience thematic series entitled "Data-Driven Multicellular Systems Biology."

Methods

Are the methods appropriate to the aims of the study, are they well described, and are necessary controls included? Choose an item.

Conclusions

Are the conclusions adequately supported by the data shown? Choose an item.

Reporting Standards

Does the manuscript adhere to the journal’s guidelines on minimum standards of reporting? Choose an item.

Choose an item.

Statistics

Are you able to assess all statistics in the manuscript, including the appropriateness of statistical tests used? Choose an item.

Quality of Written English

Please indicate the quality of language in the manuscript: Choose an item.

Declaration of Competing Interests

Please complete a declaration of competing interests, considering the following questions:

- Have you in the past five years received reimbursements, fees, funding, or salary from an organisation that may in any way gain or lose financially from the publication of this manuscript, either now or in the future?
- Do you hold any stocks or shares in an organisation that may in any way gain or lose financially from the publication of this manuscript, either now or in the future?
- Do you hold or are you currently applying for any patents relating to the content of the manuscript?
- Have you received reimbursements, fees, funding, or salary from an organization that holds or has applied for patents relating to the content of the manuscript?
- Do you have any other financial competing interests?
- Do you have any non-financial competing interests in relation to this paper?

If you can answer no to all of the above, write 'I declare that I have no competing interests' below. If your reply is yes to any, please give details below.

I declare that I have no competing interests.

I agree to the open peer review policy of the journal. I understand that my name will be included on my report to the authors and, if the manuscript is accepted for publication, my named report including any attachments I upload will be posted on the website along with the authors' responses. I agree for my report to be made available under an Open Access Creative Commons CC-BY license (http://creativecommons.org/licenses/by/4.0/). I understand that any comments which I do not wish to be included in my named report can be included as confidential comments to the editors, which will not be published.

Choose an item.

To further support our reviewers, we have joined with Publons, where you can gain additional credit to further highlight your hard work (see: https://publons.com/journal/530/gigascience). On publication of this paper, your review will be automatically added to Publons, you can then choose whether or not to claim your Publons credit. I understand this statement.

Yes Choose an item.
