# Supplementary material for: High-throughput microscopy reveals the impact of multifactorial environmental perturbations on colorectal cancer cell growth
Source: Gigascience. 2021 Apr 19;10(4):giab026. doi: 10.1093/gigascience/giab026 (PMC8054261; doi:10.1093/gigascience/giab026)
Supplement: giab026_Reviewer_2_Report_Original_Submission — Ramya Sivakumar -- 6/16/2020 Reviewed [file giab026_reviewer_2_report_original_submission.pdf]

**Reviewer Report**

**Title: High throughput microscopy reveals the impact of multifactorial environmental perturbations on colorectal cancer cell growth**

**Version: Original Submission** **Date:** 6/16/2020

**Reviewer name: Ramya Sivakumar**

**Reviewer Comments to Author:**

The article is simple, straightforward and easy to follow. The ability of high content screening to measure phenotypic changes with alterations to the tumor microenvironment such as hypoxia and matrix stiffness has been studied using 3 different cell lines. In addition to modifying oxygen levels, the authors also perform co-cultures with an interesting acellular liver ECM disk to observe the effect of liver scaffolds in tumor growth patterns.
While figures 1-2 report changes in multiple cell lines, figure 4 highlights the effect of liver scaffolds on only the most aggressive and metastatic cell line HCT116. Reporting the effect of liver scaffold on the growth of less aggressive and metastatic cell lines and comparing and contrasting the differences would have been more effective at studying ECM based regulation of tumor growth. The authors could also have utilized more fluorescent markers in addition to DAPI to determine the effect of the hypoxia and liver ECM on tumor biology.
Did the authors test any more chemotherapeutic drugs other than oxaliplatin ? And, what about testing combinations of drugs under these culture conditions ?
Were there plans to implant the cell lines grown in co-cultures or normoxic conditions in mice to follow tumor growth ?
Fig 3B (what's the purpose of the yellow arrow mark?)

**Methods**

Are the methods appropriate to the aims of the study, are they well described, and are necessary controls included? Choose an item.

**Conclusions**

Are the conclusions adequately supported by the data shown? Choose an item.

**Reporting Standards**

Does the manuscript adhere to the journal's guidelines on minimum standards of reporting? Choose an item.

Choose an item.

**Statistics**

Are you able to assess all statistics in the manuscript, including the appropriateness of statistical tests used? Choose an item.

**Quality of Written English**

Please indicate the quality of language in the manuscript: Choose an item.

**Declaration of Competing Interests**

Please complete a declaration of competing interests, considering the following questions:

- Have you in the past five years received reimbursements, fees, funding, or salary from an organisation that may in any way gain or lose financially from the publication of this manuscript, either now or in the future?
- Do you hold any stocks or shares in an organisation that may in any way gain or lose financially from the publication of this manuscript, either now or in the future?
- Do you hold or are you currently applying for any patents relating to the content of the manuscript?
- Have you received reimbursements, fees, funding, or salary from an organization that holds or has applied for patents relating to the content of the manuscript?
- Do you have any other financial competing interests?
- Do you have any non-financial competing interests in relation to this paper?

If you can answer no to all of the above, write 'I declare that I have no competing interests' below. If your reply is yes to any, please give details below.

I declare that I have no competing interests

I agree to the open peer review policy of the journal. I understand that my name will be included on my report to the authors and, if the manuscript is accepted for publication, my named report including any attachments I upload will be posted on the website along with the authors' responses. I agree for my report to be made available under an Open Access Creative Commons CC-BY license (http://creativecommons.org/licenses/by/4.0/). I understand that any comments which I do not wish to be included in my named report can be included as confidential comments to the editors, which will not be published.

Choose an item.

To further support our reviewers, we have joined with Publons, where you can gain additional credit to further highlight your hard work (see: https://publons.com/journal/530/gigascience). On publication of this paper, your review will be automatically added to Publons, you can then choose whether or not to claim your Publons credit. I understand this statement.

Yes Choose an item.
