# Supplementary material for: High-throughput microscopy reveals the impact of multifactorial environmental perturbations on colorectal cancer cell growth
Source: Gigascience. 2021 Apr 19;10(4):giab026. doi: 10.1093/gigascience/giab026 (PMC8054261; doi:10.1093/gigascience/giab026)
Supplement: giab026_Reviewer_3_Report_Original_Submission — Jessica Bauer -- 6/22/2020 Reviewed [file giab026_reviewer_3_report_original_submission.pdf]

Reviewer Report

Title: High throughput microscopy reveals the impact of multifactorial environmental perturbations on colorectal cancer cell growth

Version: Original Submission **Date:** 6/22/2020

Reviewer name: Jessica Bauer

Reviewer Comments to Author:

The manuscript High throughput microscopy reveals the impact of multifactorial environmental perturbations on colorectal cancer cell growth and shows that HCT116 cell growth is affected by oxygen, substrate stiffness, and liver ECM. This data set could be set for training and testing data driven computational models of cancer and other multicellular systems.
The manuscript is well written however, there are some major issues I have with the manuscript:

1. There is no stiffness mention in the background. It is only mentioned briefly in one sentence, but it is essential for your results and it is in the conclusion of the abstract. There should be at least a paragraph about stiffness in the background.
2. Why using 0.2kPa and 3GPa in the experiments? Those two values are not physiological stiffness. It would be useful to do those experiments with physiological values for example the kPa of a tumor and a normal cell and the 3GPa.
3. Discussion needs further editing.
4. HT-29 are MSS and HCT116 are MSI. There is some literature about hypoxia and MSI which could be discussed and mentioned in the background
5. Figure 1 to figure 2 needs a better transfer why the author is doing this experiment.
6. To really say that this data set could be used for training and testing data driven computational models of cancer and other multicellular systems it would need more than 3 cell lines to perform these experiments. Also, some human data would be useful to make this statement.

Methods

Are the methods appropriate to the aims of the study, are they well described, and are necessary controls included? Choose an item.

Conclusions

Are the conclusions adequately supported by the data shown? Choose an item.

Reporting Standards

Does the manuscript adhere to the journal's guidelines on minimum standards of reporting? Choose an item.

Choose an item.

Statistics

Are you able to assess all statistics in the manuscript, including the appropriateness of statistical tests used? Choose an item.

Quality of Written English

Please indicate the quality of language in the manuscript: Choose an item.

Declaration of Competing Interests

Please complete a declaration of competing interests, considering the following questions:

- Have you in the past five years received reimbursements, fees, funding, or salary from an organisation that may in any way gain or lose financially from the publication of this manuscript, either now or in the future?
- Do you hold any stocks or shares in an organisation that may in any way gain or lose financially from the publication of this manuscript, either now or in the future?
- Do you hold or are you currently applying for any patents relating to the content of the manuscript?
- Have you received reimbursements, fees, funding, or salary from an organization that holds or has applied for patents relating to the content of the manuscript?
- Do you have any other financial competing interests?
- Do you have any non-financial competing interests in relation to this paper?

If you can answer no to all of the above, write 'I declare that I have no competing interests' below. If your reply is yes to any, please give details below.

I declare that I have no competing interests

I agree to the open peer review policy of the journal. I understand that my name will be included on my report to the authors and, if the manuscript is accepted for publication, my named report including any attachments I upload will be posted on the website along with the authors' responses. I agree for my report to be made available under an Open Access Creative Commons CC-BY license (http://creativecommons.org/licenses/by/4.0/). I understand that any comments which I do not wish to be included in my named report can be included as confidential comments to the editors, which will not be published.

Choose an item.

To further support our reviewers, we have joined with Publons, where you can gain additional credit to further highlight your hard work (see: https://publons.com/journal/530/gigascience). On publication of this paper, your review will be automatically added to Publons, you can then choose whether or not to claim your Publons credit. I understand this statement.

Yes Choose an item.
